# The Drosophila *bag of marbles* Gene Interacts Genetically with *Wolbachia* and Shows Female-Specific Effects of Divergence

**DOI:** 10.1371/journal.pgen.1005453

**Published:** 2015-08-20

**Authors:** Heather A. Flores, Jaclyn E. Bubnell, Charles F. Aquadro, Daniel A. Barbash

**Affiliations:** Department of Molecular Biology and Genetics, Cornell University, Ithaca, New York, United States of America; Fred Hutchinson Cancer Research Center, UNITED STATES

## Abstract

Many reproductive proteins from diverse taxa evolve rapidly and adaptively. These proteins are typically involved in late stages of reproduction such as sperm development and fertilization, and are more often functional in males than females. Surprisingly, many germline stem cell (GSC) regulatory genes, which are essential for the earliest stages of reproduction, also evolve adaptively in Drosophila. One example is the *bag of marbles* (*bam*) gene, which is required for GSC differentiation and germline cyst development in females and for regulating mitotic divisions and entry to spermatocyte differentiation in males. Here we show that the extensive divergence of *bam* between *Drosophila melanogaster* and *D*. *simulans* affects *bam* function in females but has no apparent effect in males. We further find that infection with *Wolbachia pipientis*, an endosymbiotic bacterium that can affect host reproduction through various mechanisms, partially suppresses female sterility caused by *bam* mutations in *D*. *melanogaster* and interacts differentially with *bam* orthologs from *D*. *melanogaster* and *D*. *simulans*. We propose that the adaptive evolution of *bam* has been driven at least in part by the long-term interactions between Drosophila species and *Wolbachia*. More generally, we suggest that microbial infections of the germline may explain the unexpected pattern of evolution of several GSC regulatory genes.

## Author Summary

Animals need to make gametes–sperm or eggs–in order to reproduce. Gametes are produced from a specialized tissue called the germline that is found within the testes or ovaries. These organs contain a small population of stem cells that are able to both self-renew and differentiate to generate gametes and are thus essential for maintaining gamete production throughout the reproductive lifespan of most animals. Surprisingly, some of the genes that control this process evolve rapidly between Drosophila species. We find for a key germline stem cell regulatory gene, *bag of marbles* (*bam*), that its rapid evolution affects only female but not male functions. We further report that the endosymbiont bacterium *Wolbachia* that infects insects and other species interacts with *bam* and may be contributing to the wider pattern of rapid evolution of germline stem cell regulatory genes.

## Introduction

Population genetic and comparative analyses in diverse taxa have shown that many genes involved in reproduction are evolving under adaptive evolution [1–3]. Various selective pressures have been hypothesized to drive the adaptive evolution of those reproductive genes including sexual conflict, sexual selection, pathogen resistance, and avoidance of interspecific fertilization [2,4,5]. While population genetic and comparative approaches have been valuable in identifying adaptively evolving genes [4,6–11], a combination of population genetic and functional approaches is needed to identify the adaptive phenotypes and to determine the contribution of these selective pressures.

The gene *bag of marbles* (*bam*) is an intriguing example of a rapidly evolving reproduction gene, having experienced recurrent, adaptive evolution in *D*. *melanogaster* and *D*. *simulans* [12,13]. Unlike many other reproductive genes that have experienced positive selection, however, *bam* functions early in gametogenesis, making it unlikely that many of the selective pressures mentioned above could act on it. Surprisingly, genes involved in germ cell development and cystoblast division are over-represented genome-wide among those adaptively evolving in both *D*. *melanogaster* and *D*. *simulans* [7,14].

*bam* regulates germline stem cell (GSC) differentiation and germline cyst development in both males and females. GSCs are present in a niche environment that is required to maintain their stem cell state [15,16]. When a stem cell asymmetrically divides, the daughter cell, a cystoblast, moves away from the niche, which relieves repressive mechanisms and allows it to differentiate [15–17]. The cystoblast then undergoes four synchronous mitotic divisions to generate an interconnected, 16-cell cyst. In females, one of these cells will become the oocyte and enter meiosis while the remaining 15 nurse cells will become polyploid and provide nutrients to the oocyte. In males, all 16 cells will enter meiosis and give rise to mature sperm [18].

**Fig 1:**
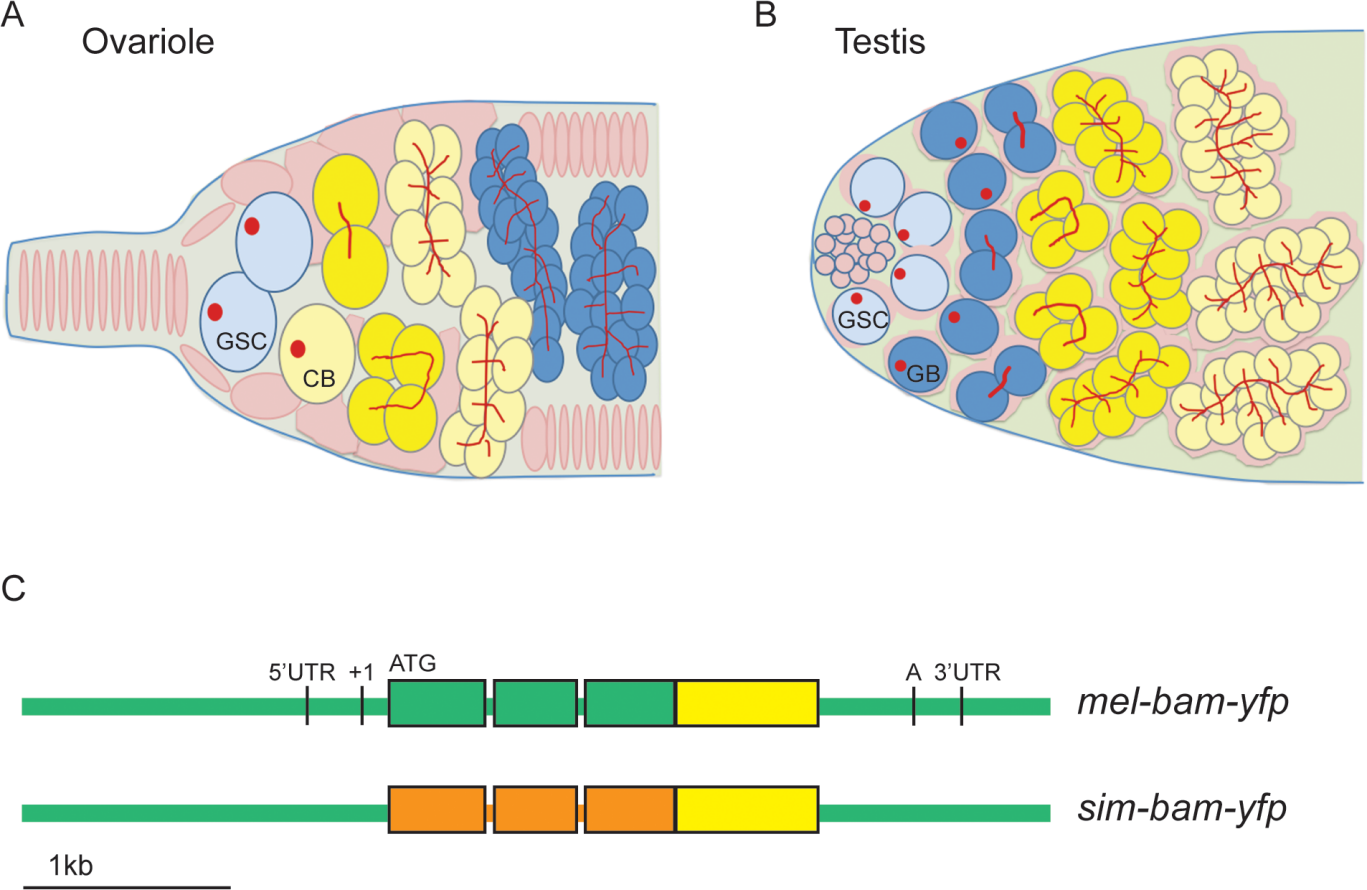
*bam* transgenic constructs. (A) Diagrams of ovariole tip and (B) testis tip of wildtype flies. GSCs differentiate into cystoblasts (CB, ovariole) or gonialblasts (GB, testis), which undergo four synchronous, mitotic divisions. In females, Bam expression (yellow) is restricted to the CB, 2-,4-, and 8-cell cysts. In males, Bam expression occurs in 4-,8-, and 16-cell cysts. Somatic cells/somatic stem cells are shown in pink, germ cells in blue and yellow (when expressing Bam), GSCs in light blue, and spectrosomes (in GSCs) and fusomes (in cysts) in red. (C) *bam* transgenic constructs. All constructs are drawn to scale and contain the entire *bam* open reading frame (thick bars), 2 small introns, and non-coding regions (thin bars). Green color corresponds to *D*. *melanogaster* sequences, orange to *D*. *simulans* sequences, and yellow to the YFP coding sequence. ATG denotes the start codon, and 5’ and 3’ UTR sequence boundaries are from *D*. *melanogaster* genome release v. 5.30 (Flybase) [106]. The transcription start site is denoted as +1 [21] and the poly(A) addition sequence is denoted as A [23].

In females, *bam* is the key factor for inducing GSCs to differentiate and is thus transcriptionally repressed in the GSC and derepressed in the cystoblast [19–21]. Bam expression is transient, as its protein is present only in late cystoblasts, and 2-, 4-, and 8-cell cysts (Fig 1A) [22]. In males, *bam* is not required for GSC differentiation, as *bam* mutant GSCs differentiate but continue undergoing mitotic divisions and never enter meiosis [23–25]. As in females, Bam protein is expressed transiently in males, as it is present only in 4-, 8-, and 16-cell cysts (Fig 1B) [25].

Bam also functions downstream of GSC differentiation in both males and females. Bam also localizes to the fusome, an ER-like organelle that interconnects the cells of a cyst, mediates the synchrony of the mitotic divisions, and likely determines the future oocyte [22,26]. This localization requires the gene *benign gonial cell neoplasm* (*bgcn)* [27], and *bam* mutants show a reduction in fusome vesicles [22]. Bam also has a role in counting cyst divisions in females [22,28,29]. This function is more clearly established in males, where the accumulation of Bam to a critical threshold is required for cysts to cease mitotic divisions and initiate spermatocyte differentiation [25,30].

The molecular function of *bam* is not fully understood, but Bam physically interacts with and requires the function of *bgcn* [27,31–33] and *Sex lethal* (*Sxl*) [34–36] in GSC differentiation in females. Sxl has been shown to bind *nanos* mRNA, downregulating it and allowing for GSC differentiation [34–36]. Additionally, Bgcn is related to the DExH-box family of ATP-dependent RNA helicases, leading to the hypothesis that Bgcn functions together with Bam to repress translation [31]. This has been shown directly in males for the target gene *mei-P26* [30].

Because *bam* is essential for fertility yet is involved in the early stages of reproduction, theories of sexual conflict and sexual selection that apply to many other rapidly evolving reproductive genes do not readily explain the adaptive evolution of *bam*. We therefore explore here interactions between *bam* and the bacterial endosymbiont, *Wolbachia pipientis*. *Wolbachia* is maternally inherited and manipulates host reproduction in a variety of organisms [37–40]. One report found that *Wolbachia* infection partially rescues the oogenesis defects of *Sxl* mutants in *Drosophila melanogaster* [41,42]. This result is an important motivation for examining possible interactions between *Wolbachia* and *bam* because a subsequent study showed that *bam* requires *Sxl* to function in GSC differentiation [34].

*Wolbachia* localization and activity are highly dynamic among Drosophila species and are controlled by both host and bacteria [43–48]. For example, in *D*. *melanogaster*, *Wolbachia* is present throughout the germline of females but preferentially accumulates at the somatic stem cell niche, a microenvironment required to maintain somatic stem cells that, when differentiated, produce follicle cells [49]. In contrast, *Wolbachia* preferentially localizes to the germline stem cell niche in *D*. *mauritiana* [46,49]. Transinfection and introgression studies have shown this trait to be primarily controlled by *Wolbachia* strain, rather than host background [48]. *Wolbachia* can rapidly spread through a population using a reproductive manipulation known as cytoplasmic incompatibility (CI), where *Wolbachia* causes the death of offspring from matings of *Wolbachia*-infected fathers with uninfected mothers [39]. When CI-inducing *Wolbachia* from *D*. *simulans* are transferred to *D*. *melanogaster*, their ability to induce CI decreases dramatically [43]. Conversely, when strains that do not induce strong CI in *D*. *melanogaster* were transinfected into *D*. *simulans*, they induced high levels of CI [50]. Additionally, some strains of *Wolbachia* do not cause CI, suggesting that both *Wolbachia* and its host control the occurrence/penetrance of CI [50].

These studies suggest that *Wolbachia* may be inducing species-specific adaptations, yet no studies to our knowledge have identified host genes that are candidates for mediating an adaptive response to *Wolbachia*. The critical function of *bam* in GSC differentiation and the striking consequences of *bam* divergence in females that we document in this study motivated us to explore interactions between *Wolbachia* and *bam*.

## Results

### Transgenic constructs to test for interspecific rescue of *D*. *melanogaster bam* mutants

To identify the functional consequences of *bam*’s divergence, we developed a transgenic system to assay the ability of a *bam* ortholog from *D*. *melanogaster* or *D*. *simulans* to rescue the female and male sterility of a *D*. *melanogaster bam* mutant. We generated strains of *D*. *melanogaster* containing transgenic copies of either *D*. *melanogaster bam* (*mel-bam-yfp*) or *D*. *simulans bam* (*sim-bam-yfp*) (Fig 1C). Each *bam* ortholog was C-terminally tagged with Yellow fluorescent protein (YFP) and driven by the native *D*. *melanogaster* regulatory region which has been previously defined [21,23]. This approach was designed in an effort to attribute any phenotypic differences to coding sequence divergence. Each transgene was integrated separately in the same position of the *D*. *melanogaster* genome at two different *attP* sites on chromosome 2 (attP16a or attP40), and then crossed into a *D*. *melanogaster bam* transheterozygous, null mutant background. PCR using primers designed to the *Wolbachia wsp* gene confirmed that *Wolbachia* was not present in the transgenic or *bam* mutant stocks (see Materials and Methods). The nomenclature used throughout this study is described in Table 1.

**Table 1:** Nomenclature.

Nomenclature	Genotype	Description
*D*. *melanogaster bam* heterozygote	Females: *bam* ^Δ59^/+	*D*. *melanogaster* with only a single wildtype copy of *bam*.
	Males: *bam* ^BG^/+	
*mel-bam-yfp*; *bam* ^-^, or *sim-bam-yfp*; *bam* ^-^	Females: *w*; φ{*w* ^+^, *transgene*}/+; *bam* ^Δ86^/*bam* ^Δ59^ (footnote a)	A single copy of a transgene in a *D*. *melanogaster bam* null mutant background. See Materials and Methods for description of different *bam* alleles.
	Males: *w*; φ{*w* ^+^, *transgene*}/+; *bam* ^Δ86^/*bam* ^BG^	
2x *mel-bam-yfp*; *bam* ^-^, or2x *sim-bam-yfp*; *bam* ^-^	Females: *w*; φ{*w* ^+^, *transgene*}, φ{*w* ^+^, *transgene*}/+ +; *bam* ^Δ86^/*bam* ^Δ59^	Two copies of a transgene in a *D*. *melanogaster bam* null background.
2x *sim-bam-yfp*; *bam* ^-^/+	Females: *w*; φ{*w* ^+^, *transgene*}, φ{*w* ^+^, *transgene*}/+ +; *bam* ^Δ59^/*+*	Two copies of *sim-bam-yfp* in a *D*. *melanogaster bam* heterozygous background. These flies are siblings of 2x *sim-bam-yfp*; *bam* ^-^ flies.
*bam-α; bam* ^-^	Females: *w; P{ry+*, *bam-α}*,*bam* ^*Δ86*^ */bam* ^*Δ59*^	A single copy of a *bam* transgene [23] in a *bam* null background.
	Males: *w; P{ry+*, *bam-α}*,*bam* ^*Δ86*^ */bam* ^*BG*^	
*bam +w*Mel	*w; bam* ^BG^/*bam* ^Δ59^ +*w*Mel	*D*. *melanogaster bam* hypomorph infected with *Wolbachia* strain *w*Mel.
*bam*-Tet	*w; bam* ^BG^/*bam* ^Δ59^	*D*. *melanogaster bam* hypomorph without *Wolbachia*.

^a^ All experiments in females designated as *mel-bam-yfp*; *bam* ^-^ were performed with this genotype. As an additional control *mel-bam-yfp*; *bam* ^Δ86^/*bam* ^BG^ (full genotype *w*; φ{*w* ^+^, *mel-bam-yfp*}/+; *bam* ^Δ86^/*bam* ^BG^) was assayed for expression level in females in Fig 2A.

**Fig 2:**
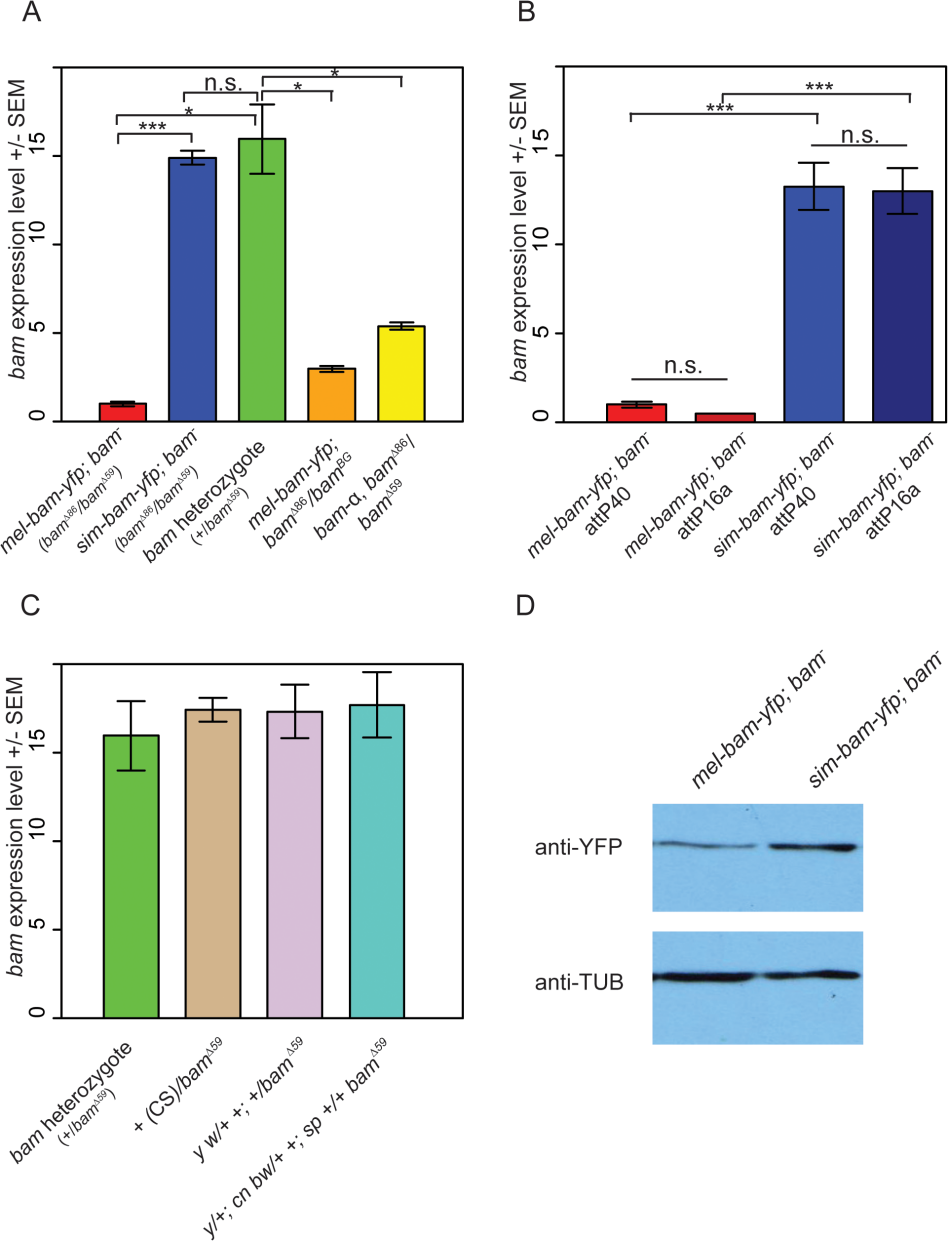
Analysis of *bam* RNA and protein expression in transgenic lines and control strains. (A) Underexpression of *bam* RNA in *mel-bam-yfp; bam* ^−^ovaries is not due to genetic background or the YFP tag. Ovarian *bam* RNA levels from *mel-bam-yfp* (red) and *sim-bam-yfp* (blue) in the *bam* mutant background (*bam* ^*Δ86*^ */bam* ^*Δ59*^), and the *D*. *melanogaster bam* heterozygote (*+*/*bam* ^*Δ59*^, green). *bam* levels of *mel-bam-yfp* in a different *bam* mutant background (*bam* ^*Δ86*^ */bam* ^*BG*^) (orange) and of a different *bam* transgene (yellow, *bam*-α; *bam* ^*–*^) are also reduced relative to the *bam* heterozygote. ΦC31-integrated transgenes in (A) are docked in attP40. (B) Transgene expression is stable across different insertion sites. We compared *bam* RNA levels from *mel-bam-yfp; bam* ^−^and *sim-bam-yfp; bam* ^−^ovaries in two different insertion sites, attP40 and attP16a. The *bam* ^−^genotype is *bam* ^Δ86^/*bam* ^Δ59^ as explained in Table 1. (C) *bam* expression levels show little variation across strains. *bam* RNA levels were compared between the *D*. *melanogaster bam* heterozygote shown in (A) to that of various wildtype or marker lines (Canton S [CS], *y w*, and *y; cn bw; sp*) that were made heterozygous over a *D*. *melanogaster bam* mutant (*bam* ^*Δ59*^). The *bam* sequence in *mel-bam-yfp* was cloned from *y; cn bw; sp*. For A-C, N = 3 biological replicates for each sample. Significance was determined by *t*-test, * *P*<0.05, **P<0.01, ***P<0.001. No significant expression differences were found in (C). (D) Western blot comparing sim-Bam-YFP and mel-Bam-YFP levels. 20μg of total protein was loaded into each lane. Western blot probed with anti-YFP or anti-α-Tubulin antibodies.

qRT-PCR analyses from ovarian cDNA provided two unexpected results. First, *bam* expression levels in *mel-bam-yfp; bam* ^*−*^(*mel-bam-yfp/+; bam* ^*Δ86*^ */bam* ^*Δ59*^, see Table 1) ovaries are 13–15-fold less than in controls with a single *D*. *melanogaster bam* allele (*bam* heterozygote of *bam* ^*Δ59*^/*+*) generated from the same cross (Fig 2A). To determine if the unexpectedly low *bam* expression in *mel-bam-yfp; bam* ^*−*^is due to a mutation caused during transformation or to a background effect, additional qRT-PCR was performed in which we found that the results are consistent in different *bam* mutant backgrounds (Fig 2A) and across different transgene insertion sites (Fig 2B). We also determined that *bam* expression in the stock from which the *bam* allele in *mel-bam-yfp* was cloned is similar to the *D*. *melanogaster* heterozygote (+/*bam* ^*Δ59*^), demonstrating that the particular allele we chose is not defective in expression (Fig 2C). Additionally, we found that *bam* expression in the heterozygous genotype used as a reference is not an outlier as it is similar across several genetic backgrounds (Fig 2C). Finally, we compared *bam* expression in *mel-bam-yfp; bam* ^*−*^to that of another *bam* transgene, *bam-α*, previously reported to fully rescue both female and male sterility of *D*. *melanogaster bam* mutants [23]. We found that the *bam-α* transgene is similarly under-expressed relative to the *D*. *melanogaster bam* heterozygote (Fig 2A). We attempted to perform similar qRT-PCR analyses of *bam* expression in males, but could not generate reliable results due to its low level of expression. Overall, these results demonstrate that *mel-bam* transgenes do not express at a wildtype level in females. This is likely caused by the lack of some regulatory sequences, although we cannot eliminate the possibility that *bam* transgenes are particularly sensitive to position effects. We therefore designed the genetic assays below to assess whether *mel-bam-yfp* is fully functional.

The second unexpected result is that *bam* expression in *sim-bam-yfp; bam* ^*−*^(*sim-bam-yfp/+; bam* ^*Δ86*^ */bam* ^*Δ59*^, see Table 1) ovaries is similar to the *D*. *melanogaster bam* heterozygote and ~13–15-fold higher than *mel-bam-yfp*; *bam* ^*−*^(Fig 2A and 2B), despite the fact that both transgenes contain the same *D*. *melanogaster bam* regulatory region. We examined protein levels by Western blots and found that sim-Bam-YFP accumulates ~2–3-fold higher than mel-Bam-YFP which is considerably less than the difference in RNA levels (Fig 2D). We conclude that *bam* coding sequence (CDS) divergence affects both RNA and protein levels. We were unable to assess how protein levels from each transgene compare to wildtype levels as anti-Bam antibodies did not work well on Western blots under our experimental conditions (monoclonal mouse Anti-BamC, rabbit Anti-Bam) [22,51,52]. The difference in expression levels between the transgenes does complicate the ability to attribute phenotypic differences between the orthologs to coding sequence divergence. We therefore expanded our analyses to include the *D*. *melanogaster bam* heterozygote as a control, since its expression level is not significantly different from *bam* levels in *sim-bam-yfp; bam* ^*–*^, resulting in a three-way comparison: *mel-bam-yfp; bam* ^*−*^vs. *bam* heterozygote, *mel-bam-yfp; bam* ^*−*^vs. *sim-bam-yfp; bam* ^*–*^, and *sim-bam-yfp; bam* ^*−*^vs. *bam* heterozygote (See S1 Fig for crossing diagrams).

### *sim-bam-yfp* rescues the male sterility but not female sterility of *D*. *melanogaster bam* mutants

**Fig 3:**
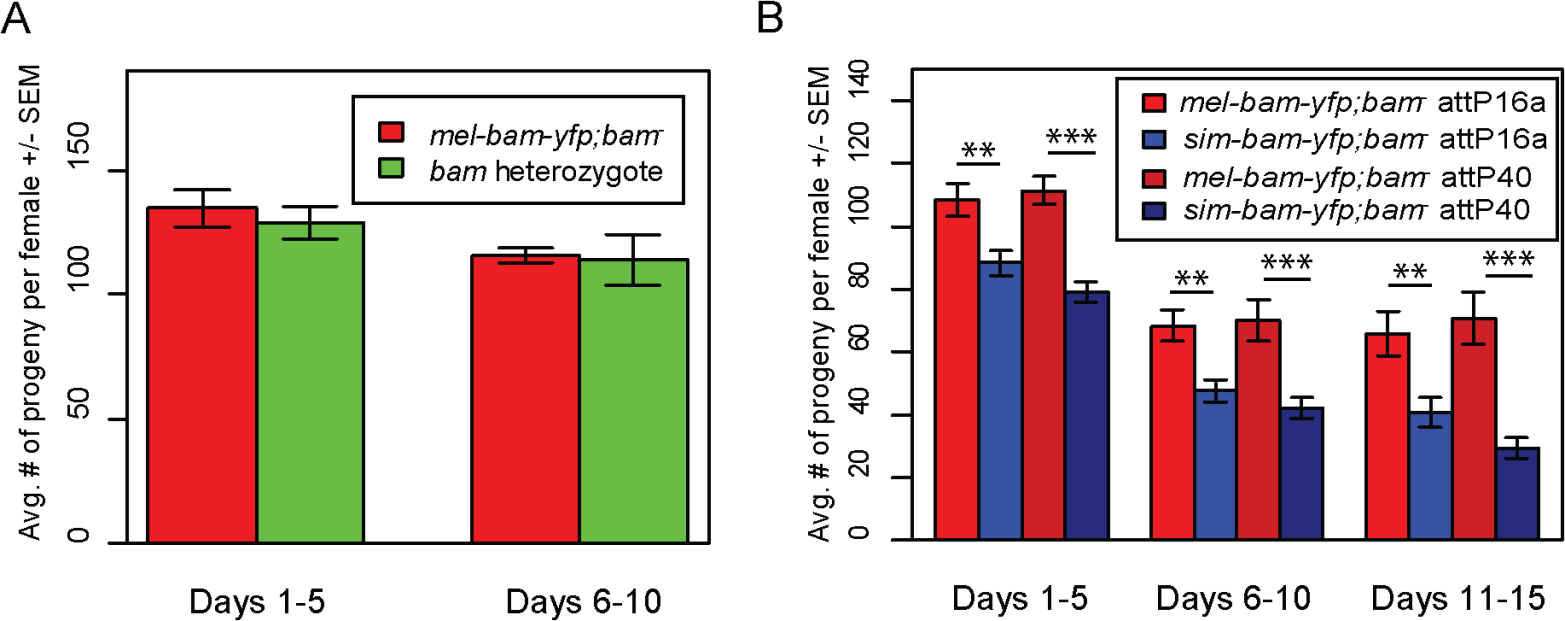
*sim-bam-yfp* does not fully rescue *D*. *melanogaster bam* mutant female sterility. One transgenic female (or heterozygous female) and two tester males were allowed to mate and the trio was transferred to a new vial every five days. Fertility is shown as the average number of progeny per female +/- SEM for each vial. (A) *mel-bam-yfp* rescues *D*. *melanogaster bam* female sterility. N ranged between 22 and 24 females at start of experiment; due to female mortality N ranged between 17 and 18 at end of experiment. (B) *sim-bam-yfp* cannot fully rescue *D*. *melanogaster bam* female sterility. *mel-bam-yfp; bam* ^−^is shown in red and compared to *sim-bam-yfp; bam* ^−^in blue. N ranged between 38 and 40 females at start of experiment; due to female mortality N ranged between 26 and 33 at end of experiment. (*t*-test, * *P*<0.05, **P<0.01, ***P<0.001).

To assay transgene function, we crossed each into a *D*. *melanogaster bam* transheterozygous, null mutant background. Sibling flies that were heterozygous for *bam* but did not carry a transgene were used as a control for comparison in fertility experiments (S1 Fig). We found that *mel-bam-yfp* fully rescues *D*. *melanogaster bam* female sterility to the level of the *D*. *melanogaster bam* heterozygous control (Fig 3A), suggesting that this transgene is fully functional in females despite having a reduced expression level relative to wildtype *bam* alleles. However, *sim-bam-yfp; bam* ^*−*^females were significantly less fertile than *mel-bam-yfp; bam* ^*−*^at every time point in the experiment for both insertion sites tested (Fig 3B), demonstrating the *sim-bam-yfp* cannot fully rescue *D*. *melanogaster bam* female sterility.

**Fig 4:**
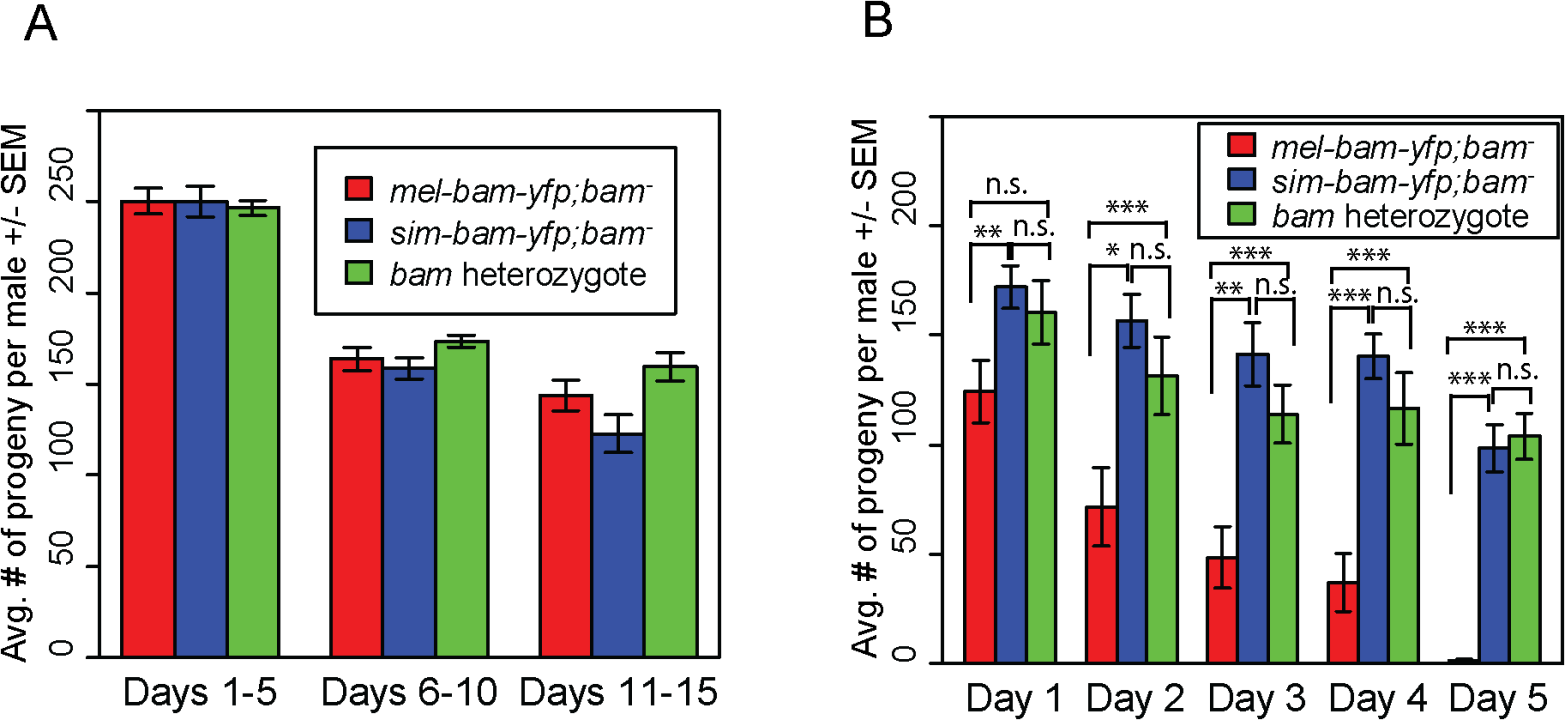
*sim-bam-yfp* rescues *D*. *melanogaster bam* mutant male sterility. (A) *mel-bam-yfp* and *sim-bam-yfp* both rescue male sterility under standard fertility conditions. One male and two tester females were allowed to mate and the trio was transferred to a new vial every five days. No comparisons are significantly different. N ranged between 42 and 46 males at start of experiment; due to mortality N ranged between 37 and 43 at end of experiment. (B) *sim-bam-yfp* but not *mel-bam-yfp* rescues male sterility under sperm exhaustion conditions. One male was allowed to mate with a new pair of virgin tester females everyday for five days. Male fertility is the average number of progeny per male +/- SEM for each vial. N ranged between 28 and 33 males at start of experiment; due to mortality N ranged between 22 and 28 at end of experiment. Transgenes are inserted in attP40. (*t*-test, *P<0.05, **P<0.01, ***P<0.001).

In contrast to female fertility assays, *sim-bam-yfp; bam* ^*−*^males were as fertile as their *mel-bam-yfp; bam* ^*−*^or *D*. *melanogaster bam* heterozygous counterparts (Fig 4A). To test for more subtle differences in male fertility, we used a sperm exhaustion mating assay by providing the males with two new, virgin females every day over a five-day period. Surprisingly, *mel-bam-yfp* does not fully rescue male sterility, suggesting that this transgene is not fully wildtype in function (Fig 4B). Under sperm exhaustion conditions *mel-bam-yfp; bam* ^*−*^males become sterile quickly which we also found when using the *bam-α* transgene previously reported to fully rescue *bam* male sterility (S2A Fig) [23], suggesting that *D*. *melanogaster bam* transgenes are unable to fully rescue male sterility. We therefore compared *sim-bam-yfp; bam* ^*−*^to *bam* heterozygotes under sperm exhaustion conditions and found that *sim-bam-yfp* fully rescues male sterility. While we were unable to accurately quantify *bam* RNA expression in males due to its low expression, we found that Bam-YFP protein expressed from both transgenes localizes in testes (S2B and S2C Fig) in a manner similar to published reports [25]. These data demonstrate that *sim-bam* divergence strongly affects females yet causes no observable defects in males.

### Ovaries from *sim-bam-yfp; bam* ^*−*^females show multiple defects including GSC loss but not a "bag of marbles" phenotype

**Fig 5:**
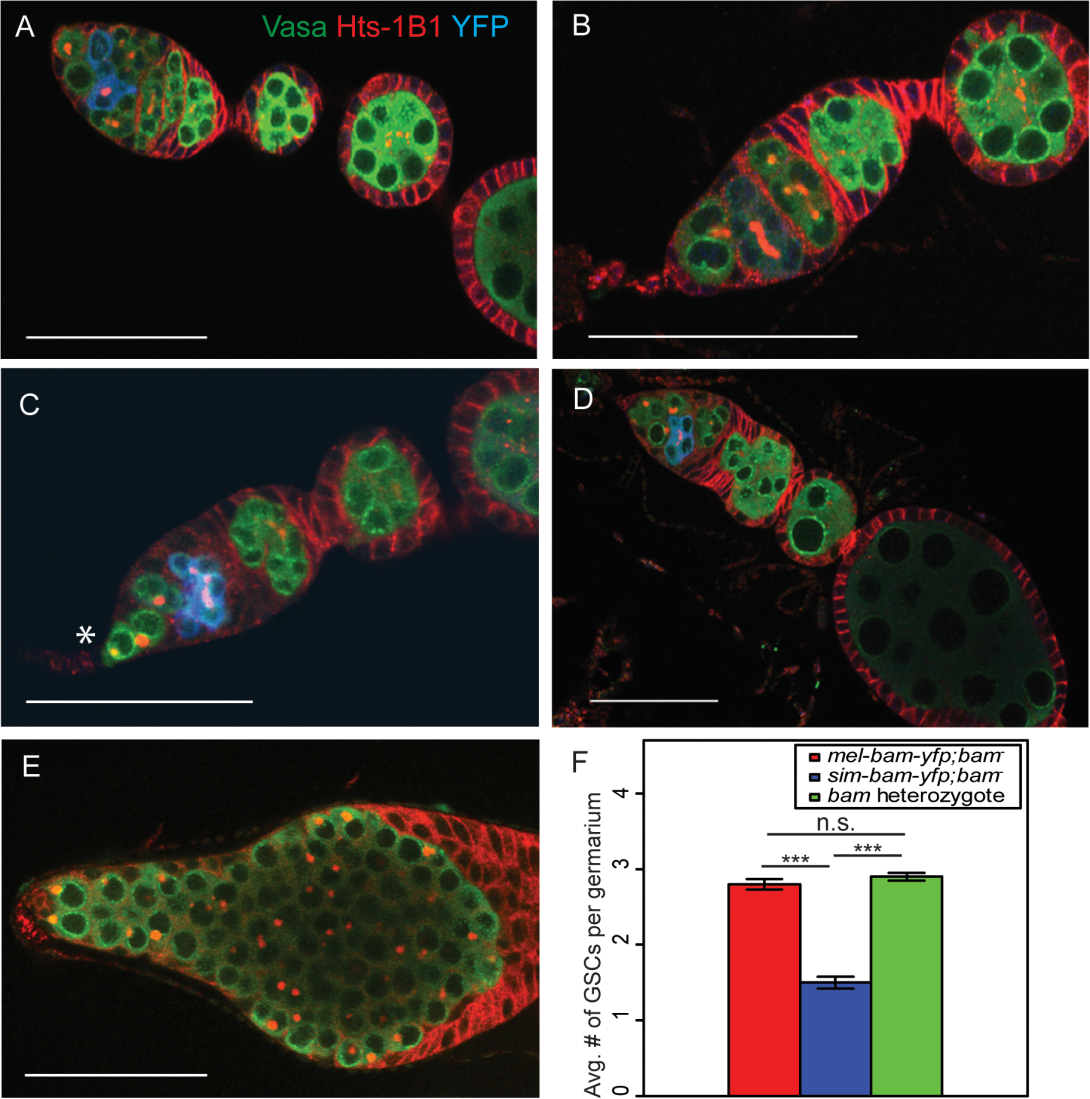
*sim-bam-yfp; bam* ^−^ovaries have multiple defects. (A-B) *mel-bam-yfp; bam* ^−^ovaries show wildtype morphology including proper Bam-YFP expression, correct number of GSCs identified by spectrosomes, and proper numbers of cells/cyst. (C-D) *sim-bam-yfp; bam* ^−^ovaries show reduced number of GSCs (*) and contain egg chambers with improper number of cells/cyst. (E) *D*. *melanogaster bam* null mutant shows “bag of marbles” phenotype. (A-E) Ovaries are from flies aged 3–5 days post-eclosion and stained with antibodies to Vasa (green), Hts-1B1 (red), and YFP (blue). Scale bar, 50μm. (F) Average GSC number across different genotypes. N = 50 ovarioles. (*t*-test, ***P<0.001).

To determine the cause of the reduced fertility of *sim-bam-yfp; bam* ^*−*^females, we stained *mel-bam-yfp; bam* ^*−*^and *sim-bam-yfp; bam* ^*−*^ovaries with antibodies to the germline marker Vasa, the fusome marker Hts-1B1, and the YFP tag in Bam-YFP. The ovaries of flies with *mel-bam-yfp; bam* ^*−*^show wildtype morphology (Fig 5A and 5B). GSCs were identified by their spherical fusome (i.e. the spectrosome) and their location within the germarium. *mel-bam-yfp*; *bam* ^*−*^ovaries had 2–3 GSCs per germarium, which is comparable to wildtype levels, and Bam was properly localized [22,53]. Furthermore, the vast majority of egg chambers underwent the proper number of cyst divisions giving rise to 16-cell cysts (S1 Table). In contrast, ovaries from *sim-bam-yfp; bam* ^*−*^flies showed multiple ovarian defects that increased as the flies aged (Fig 5C and 5D). First, they exhibit stem cell loss, with an average of only 1.5 GSCs per ovariole when young (days 1–5; Fig 5F). Second, as the flies age (days 6–15) they appeared to have a reduction in the number of ovarioles containing mature egg chambers as a consequence of GSC loss, though we did not quantify this effect. Third, many of the egg chambers (24/100) that are present have an improper number of cyst divisions and show mitotic synchrony defects (S1 Table). Mitotic synchrony defects are typically seen with fusome mutants (e.g. *hts* [54] and *α-spectrin* [55]) suggesting that *sim-bam-yfp; bam* ^*−*^flies may have fusome defects. However, *sim-bam-yfp; bam* ^*−*^ovaries have both reduced and increased numbers of cyst divisions while fusome mutants have only reduced numbers, suggesting instead that *sim-bam-yfp* cannot properly regulate the number of cyst divisions, independently of potential fusome defects. Despite these multiple ovarian defects, it is important to note that sim-Bam-YFP shows a proper localization pattern (Fig 5C and 5D). It is absent in GSCs and present in mitotically active cysts, suggesting that the defects are not due to gross misregulation of Bam. Furthermore, *sim-bam-yfp; bam* ^*−*^flies never show the *D*. *melanogaster bam* null mutant phenotype of tumorous ovaries [23] (e.g see Fig 5E), suggesting that *sim-bam-yfp* is capable of rescuing the GSC differentiation defect in *D*. *melanogaster bam* mutant females.

### *sim-bam-yfp; bam* ^*−*^ovarian defects are dose dependent and are partially suppressed by *D*. *melanogaster bam* ^*+*^

**Fig 6:**
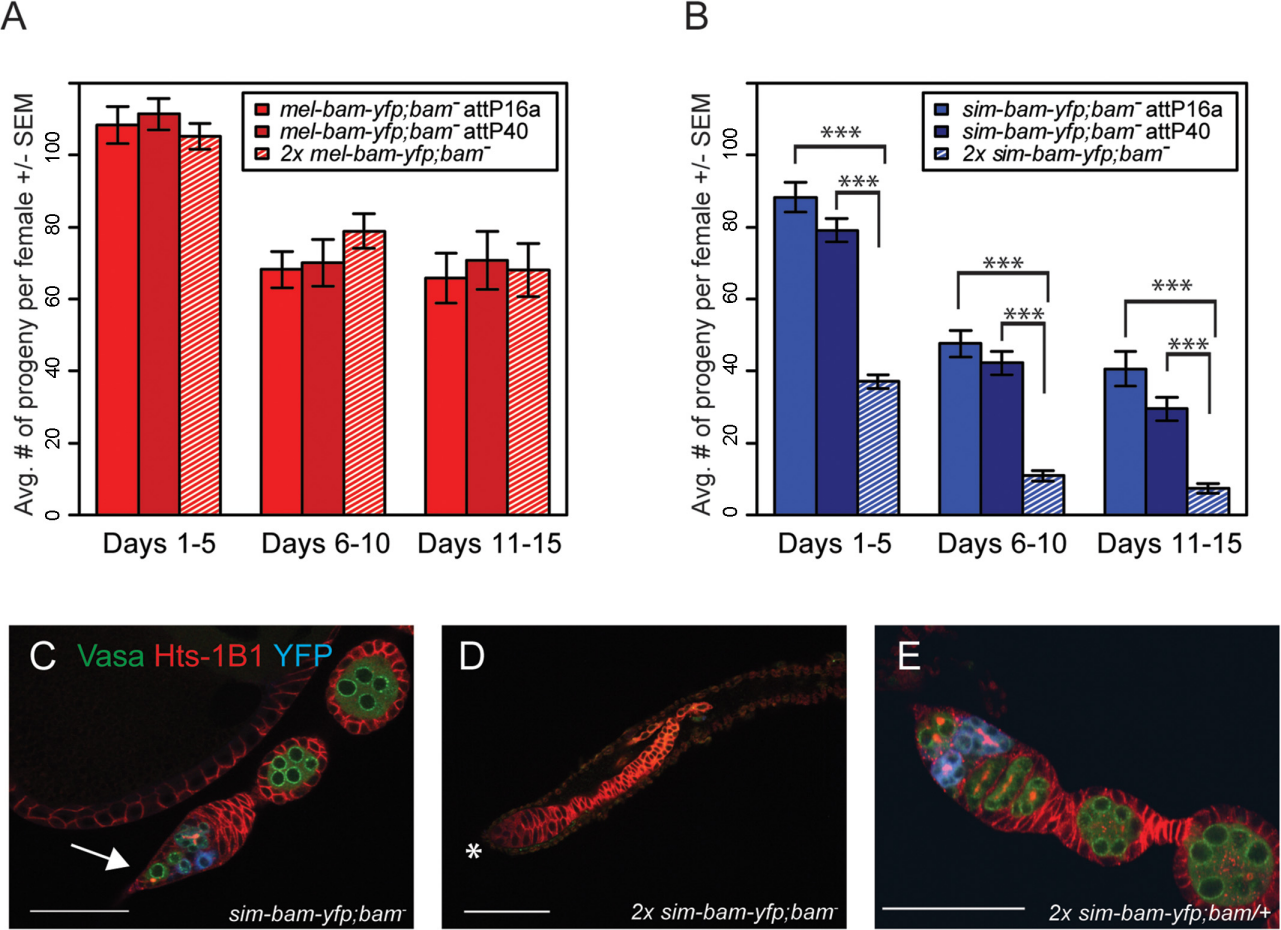
*sim-bam-yfp; bam* ^−^female fertility decreases with additional copies of *sim-bam-yfp*. (A, B) Fertility comparison of *bam* ^*−*^flies with one versus two copies of *mel-bam-yfp* (A) or *sim-bam-yfp* (B). For A and B, one female and two tester males were allowed to mate and the trio was transferred to a new vial every five days. Fertility is shown as the average number of progeny per female +/- SEM for each vial. (A) N ranged between 38 and 40 females at start of experiment; due to female mortality N ranged between 26 and 32 at end of experiment. (B) N ranged between 36 and 40 females at start of experiment; due to female mortality N ranged between 32 and 33 at end of experiment. All *mel-bam-yfp; bam* ^−^comparisons are not significant while all *sim-bam-yfp; bam* ^−^comparisons between one and two copies are highly significant (*t*-test, ***P<0.001). (C) An ovariole from *sim-bam-yfp; bam* ^−^that has only a single GSC (arrow). (D) An ovariole from 2x *sim-bam-yfp; bam* ^−^showing a complete loss of GSCs and germline as indicated by lack of Vasa staining. Asterisk indicates anterior end of germarium where GSCs normally reside. (E) A 2x *sim-bam-yfp; bam/+* ovariole shows a more wildtype ovary morphology compared to its 2x *sim-bam-yfp; bam* ^−^sibling. For C-E, ovaries are from flies aged for 3–5 days post-eclosion and are stained for Vasa (green), Hts-1B1 (red), and YFP (blue). Scale bar, 50μm.

The above experiments suggest that *sim-bam-yfp* does not function properly in a *D*. *melanogaster* background and may be acting in a gain-of-function manner as observed by the loss of GSCs. We further explored this by asking if adding additional copies of the *mel-bam-yfp* or *sim-bam-yfp* transgenes either improve or worsen the fertility phenotypes. We found no significant differences in fertility when comparing *mel-bam-yfp; bam* ^*−*^(one transgene copy) to 2x *mel-bam-yfp; bam* ^*−*^(two transgene copies, see Table 1) (Fig 6A). However, 2x *sim-bam-yfp; bam* ^*−*^(two transgene copies) flies showed a significant decrease in fertility when compared to *sim-bam-yfp; bam* ^*−*^(one transgene copy) and were nearly sterile by day 15 (Fig 6B).

Ovarioles from 2x *sim-bam-yfp; bam* ^*−*^flies showed accelerated rates of stem cell loss, even in young (1–5 day old) flies (Fig 6D and S2 Table), as compared to *sim-bam-yfp; bam* ^*−*^(Fig 6C). They typically lacked GSCs and in some cases no longer contained any germline cells, as seen by lack of Vasa staining (Fig 6D). This phenotype contrasts with *sim-bam-yfp; bam* ^*−*^flies, where GSCs were almost always present in every ovariole though often reduced in number (see Fig 5C and 5D).

We performed qRT-PCR comparing the ovarian RNA expression levels of *bam* from flies with one or two copies of the transgene. As expected, doubling the dose of the transgenes results in an approximate doubling of expression for both *mel-bam-yfp* and *sim-bam-yfp* (S3A Fig). Notably, however, *bam* RNA levels of 2x *sim-bam-yfp; bam* ^*−*^are not greater than in *D*. *melanogaster* wildtype flies (S3A Fig). Additionally, sim-Bam in 2x *sim-bam-yfp; bam* ^*−*^ovaries does not show aberrant localization when present (S3B and S3C Fig). Thus, we conclude that the 2x *sim-bam-yfp; bam* ^*−*^defects are specifically due to increased dosage of the functionally diverged *D*. *simulans bam*, rather than to a general effect of increasing *bam* dosage or gross mislocalization.

We further explored the nature of *sim-bam-yfp*-mediated defects by asking how they are modulated in the presence of a wildtype *D*. *melanogaster bam* allele. We envisioned 3 possible outcomes. The first is that if the effects are purely due to increased dosage then they should become worse with the addition of wildtype *D*. *melanogaster bam*. The second is that if the defects are purely neomorphic as a consequence of *D*. *simulans bam* divergence, then they should be unchanged. In other words *sim-bam-yfp* will be dominant over *D*. *melanogaster bam*. And the third is that if the defects are due to a failure of *sim*-*bam* function due to divergence, then they should be rescued by *D*. *melanogaster bam* and thus be recessive.

We assayed our transgenes with the addition of an endogenous copy of *D*. *melanogaster bam*. We found that *sim-bam-yfp; bam* ^*–*^-dependent defects are mostly alleviated by the addition of even a single endogenous copy of *D*. *melanogaster bam* (Fig 6E and S2 Table). This result suggests that *D*. *melanogaster bam* is dominant over *sim-bam-yfp*, but it is unlikely that *sim-bam-yfp* is simply a loss-of-function allele as the *sim-bam-yfp; bam* ^*−*^phenotypes do not match *bam* loss-of-function alleles in *D*. *melanogaster*. We therefore suggest that *sim-bam-yfp* has both loss and gain of function attributes. Several hybrid incompatibility alleles, alleles that when expressed in a hybrid background result in sterility or lethality, show similar properties [56,57].

### *sim-bam-yfp* defects in females are not due to failure of interactions with *bgcn*

**Fig 7:**
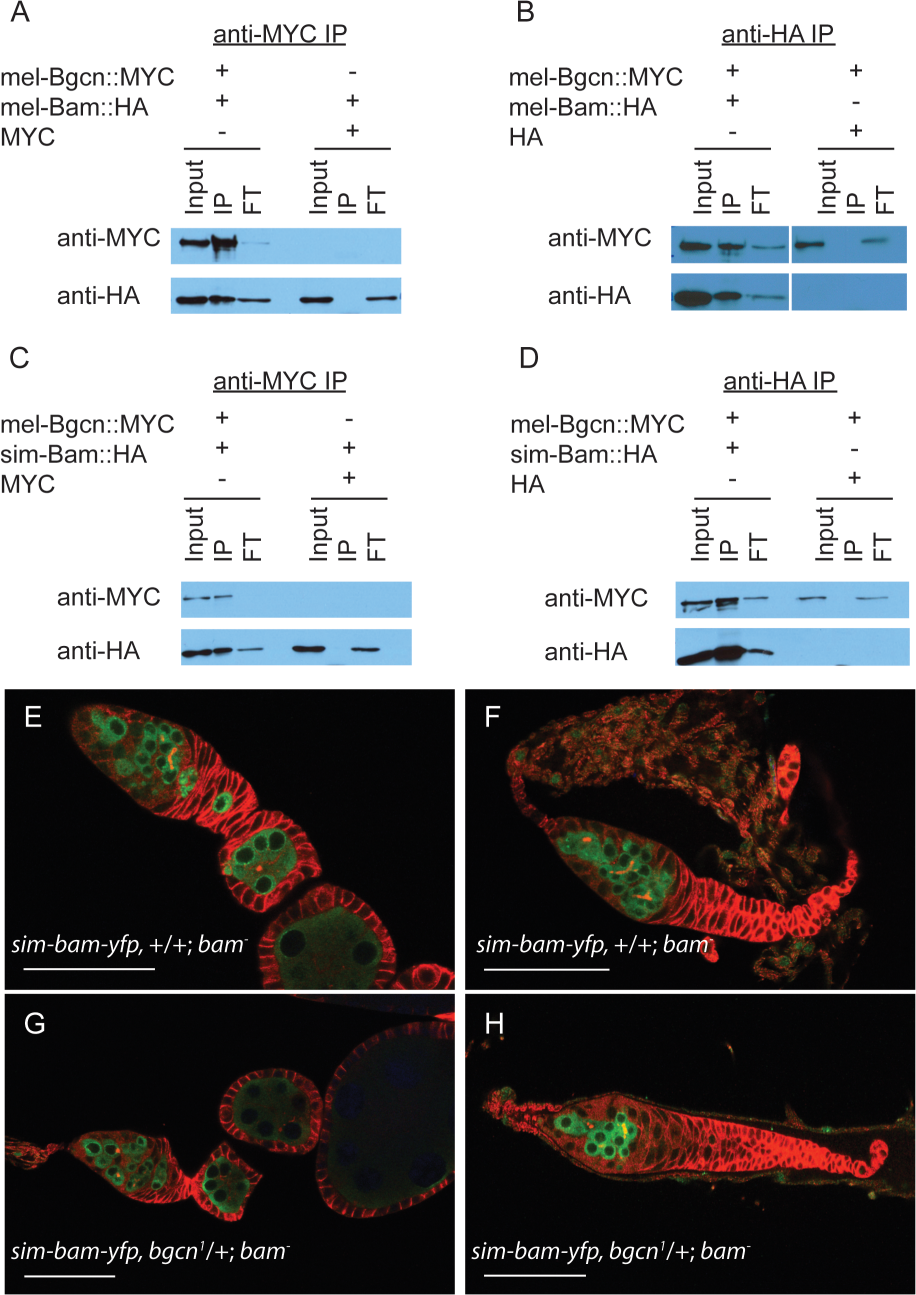
sim-Bam maintains interactions with mel-Bgcn in immunoprecipitates from S2 cells. (A-B) Control experiments with mel-Bam and mel-Bgcn. (A) Cells were transfected with mel-Bam::HA and either mel-Bgcn::MYC or MYC. Anti-MYC immunoprecipitates were analyzed by Western blot. (B) Cells were transfected with mel-Bgcn::MYC and either mel-Bam::HA or HA. Anti-HA immunoprecipitates were analyzed by Western blot. (C-D) IP experiment with sim-Bam and mel-Bgcn. (C) Cells were transfected with sim-Bam::HA and either mel-Bgcn::MYC or MYC. Anti-MYC immunoprecipitates were analyzed by Western blot. (D) Cells were transfected with mel-Bgcn::MYC and either sim-Bam::HA or HA. Anti-HA immunoprecipitates were analyzed by Western blot. Gels are loaded with 25% of total input (Input), 100% of immunoprecipitate (IP), and 10% of protein that did not immunoprecipitate (flow through, FT). (E-F) Ovaries of *sim-bam-yfp;bam* ^*−*^flies show a varying range of ovarian defects with mild (E) and moderate (F) examples shown for comparison. (G-H) Removal of a copy of *bgcn* (*bgcn* ^*1*^) does not enhance the range of phenotypes seen in *sim-bam-yfp;bam* ^*−*^ovaries. No tumorous ovaries were seen (N > 50 ovarioles). (E-H) Ovaries are stained with antibodies to Vasa (green) and Hts-1B1 (red). Scale bar, 50μm.

In *D*. *melanogaster*, Bam and Bgcn physically interact [30,32,33], and like *bam*, *bgcn* is also evolving under rapid, adaptive evolution in both *D*. *melanogaster* and *D*. *simulans* [12]. One might expect that if substitutions occurred that reduce their interaction, compensatory mutations would be selected for to re-establish a strong interaction. Therefore, independent and compensatory substitutions occurring at Bam and Bgcn within each species might render the protein partners incapable of, or less efficient at, interacting when brought together with the heterospecific protein. To determine if sim-Bam and mel-Bgcn interact with one another, we performed immunoprecipitation assays from Drosophila S2 cells. Cells were transiently transfected with either mel-Bam::HA or sim-Bam::HA, and with mel-Bgcn::MYC transgenes. We found that in reciprocal immunoprecipitation experiments both the conspecific and heterospecific Bam coimmunoprecipatated with mel-Bgcn::MYC, indicating that sim-Bam can interact with mel-Bgcn (Fig 7A–7D).

These assays involve gene over-expression and cannot discriminate whether the protein interactions are reduced in efficacy. Ohlstein et al. [31] showed that *bgcn* acts as a dominant enhancer of partial female sterility caused by *D*. *melanogaster bam* hypomorphic mutants. Reducing *bgcn* dosage exacerbated the *bam* phenotype, causing sterility and giving rise to completely tumorous ovaries. We reduced the copy number of *bgcn* by half (*bgcn* ^*1*^/+) in *sim-bam-yfp; bam* ^*−*^flies and found no exacerbation of the *sim-bam-yfp* phenotype (Fig 7E–7H). Additionally, adding a copy of *sim-bam-yfp* rescued the *bgcn*-induced sterility of the *bam* hypomorph (S4 Fig). Together the co-immunoprecipitation and genetic-interaction experiments strongly suggest that *sim-bam-yfp; bam* ^*−*^ovarian defects are not due to an inability of sim-Bam to interact with mel-Bgcn.

### *Wolbachia* infection partially rescues the sterility of *bam* hypomorphic mutants

Our transgenic rescue experiments suggest that *sim-bam-yfp* has diverged specifically in regards to its role in the female germline. The bacterial endosymbiont *Wolbachia pipentis* is maternally inherited and manipulates its host to ensure transmission [39] and could thus provide selective pressures on genes in the female germline such as *bam*. To explore possible interactions between *bam* and *Wolbachia*, we crossed a naturally occurring strain of *D*. *melanogaster Wolbachia*, *w*Mel, into a heteroallelic combination of *bam* alleles (used above in *bam* genetic-interaction assays) that results in a hypomorphic phenotype [31,32]. *bam* ^*BW*^/*bam* ^*Δ59*^ flies lacking *w*Mel *Wolbachia* are weakly fertile, giving rise to a mix of tumorous and wildtype egg chambers [31]. Thus, the number of nurse-cell positive egg chambers (i.e. non-tumorous egg chambers) can be counted to look for enhancers or suppressors of *bam* activity [31–33,58]. We compared *bam* ^*BW*^/*bam* ^*Δ59*^ flies infected with *w*Mel, denoted as "*bam* +*w*Mel", to hypomorphic flies cured of *Wolbachia* using tetracycline, denoted as "*bam*-Tet". We found that the ovarioles of *bam* +*w*Mel flies contain significantly more nurse-cell-positive egg chambers than the *bam*-Tet flies (S3 Table).

**Fig 8:**
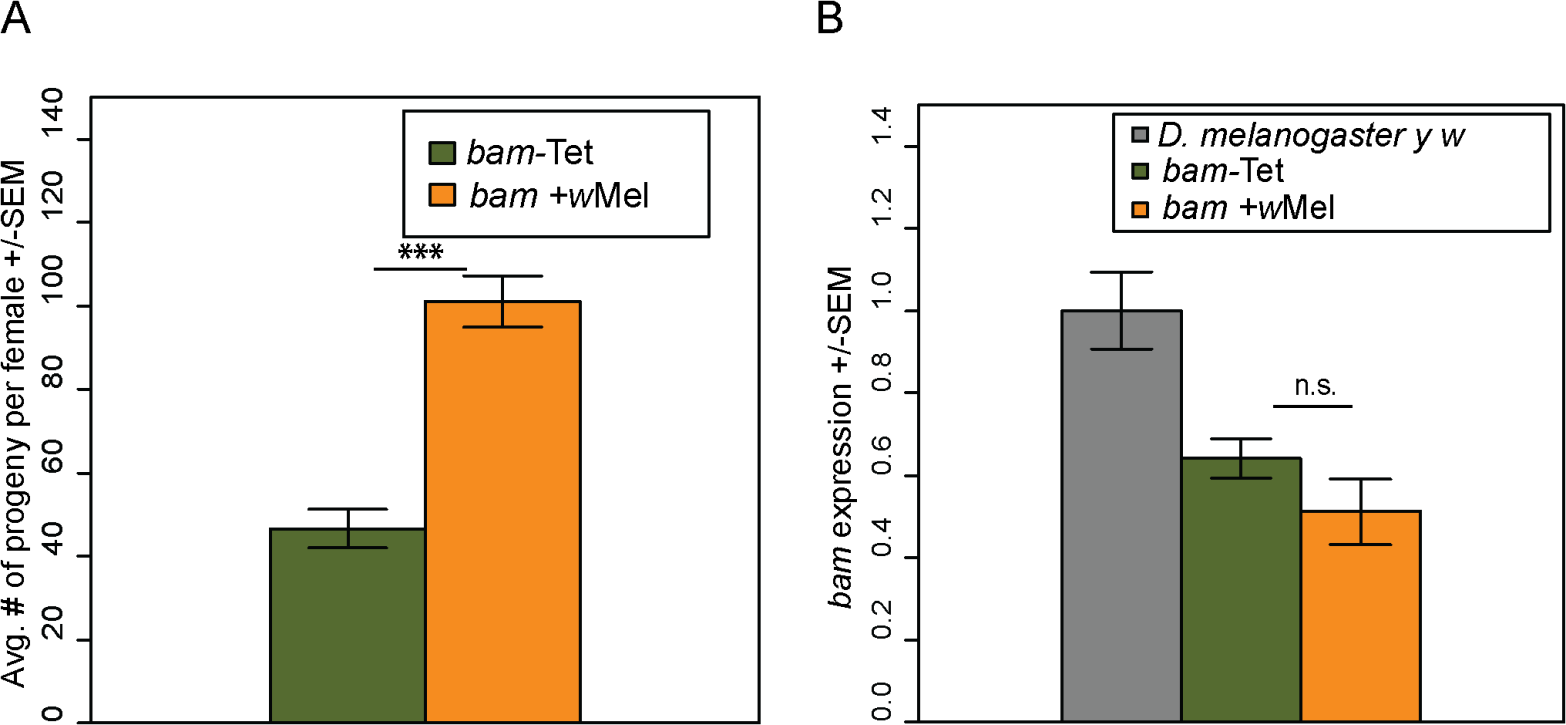
*Wolbachia* increases the fertility of *D*. *melanogaster bam* hypomorphs without altering *bam* RNA levels. (A) One female and two tester males were allowed to mate and the trio was removed from the vial after 8 days. Fertility is shown as the average number of progeny per female +/- SEM for each vial. N = 20. *Wolbachia*-infected (*w*Mel) *bam* hypomorphs are significantly more fertile than uninfected *bam* hypomorphs, *bam*-Tet (*t*-test, ***P<0.001). (B) qRT-PCR of ovarian mRNA from *D*. *melanogaster bam* hypomorphs with and without *Wolbachia*. The *D*. *melanogaster* marker strain *y w* (grey, two wildtype copies of *bam*) is shown for reference. There is no statistical difference in *bam* expression of the *bam* hypomorph with and without *Wolbachia* (*P* = 0.253; *t*-test).

We then assayed the fertility of the *bam +w*Mel *and bam*-Tet females. We found that the presence of *Wolbachia* increases the fertility of *bam* +*w*Mel females to high levels (Fig 8A; compare to Fig 3, days 1–5). The fertility increase was only observed in *bam* hypomorphs and not in combinations of *bam* null alleles that result in complete female sterility (*bam* ^*Δ86*^ */bam* ^*Δ59*^ *+w*Mel, N = 20). The fertility increase is not due to effects on *bam* mRNA levels, as expression is not significantly different between *bam* +*w*Mel and *bam*-Tet females (Fig 8B). Fertility assays were also performed in males. However, *bam* hypomorphic males were completely sterile, and the presence of *Wolbachia* had no rescuing effect (*bam* +*w*Mel, N = 20; *bam*-Tet, N = 20).

To ensure that the fertility rescue of the *bam* hypomorph was not due to a difference in the gut microbiota caused by tetracycline treatment, we repeated the experiment by controlling for the gut microbial composition (see Materials and Methods). The female fertility assay was repeated and produced very similar results showing that *bam* +*w*Mel females are significantly more fertile than *bam*-Tet females (S5 Fig). This experiment demonstrates that fertility rescue of *bam* hypomorphs is specifically due to *Wolbachia* infection.

### *Wolbachia* interacts differentially with *mel-bam-yfp* and *sim-bam-yfp*

**Fig 9:**
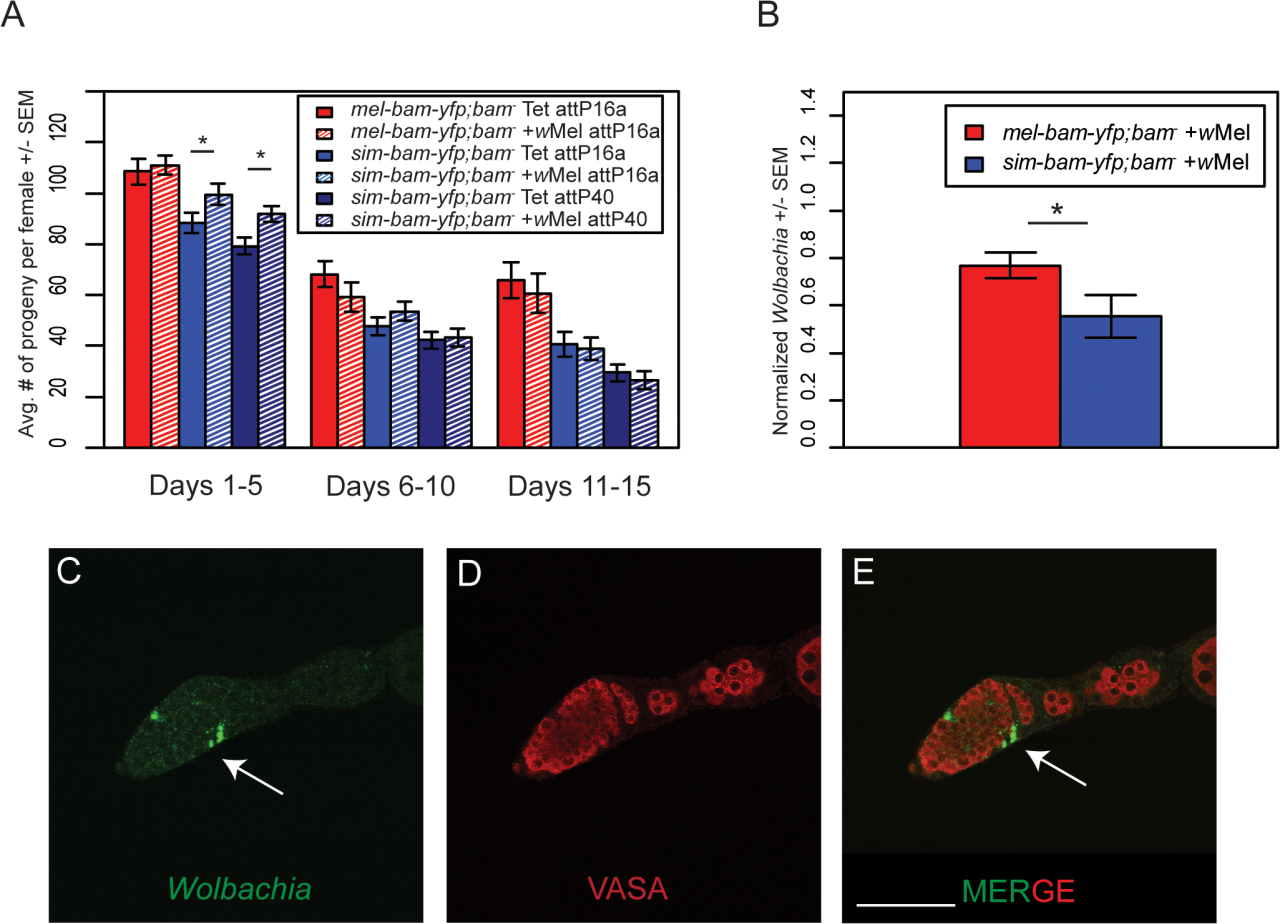
*Wolbachia* interacts with *sim-bam-yfp; bam* ^−^in females. (A) Female fertility assay. One female and two tester males were allowed to mate and the trio was transferred to a new vial every five days. Fertility is shown as the average number of progeny per female +/- SEM for each vial. (*t*-test, *P<0.05). All comparisons between *mel-bam-yfp; bam* ^−^+*w*Mel and *mel-bam-yfp; bam* ^−^Tet are not significant. All day 6–10 and 11–15 comparisons between *sim-bam-yfp*; *bam* ^−^+wMel and *sim-bam-yfp*; *bam* ^−^Tet are not significant. N ranged between 38 and 40 females at start of experiment; due to female mortality N ranged between 26 and 33 at end of experiment. (B) q-PCR for *w*Mel titer was performed from ovarian DNA from the indicated genotypes using *Wolbachia*-specific primers. (*t*-test, *P<0.05). N = 3. (C-E) *Wolbachia* localizes to the SSCN in *sim-bam-yfp; bam* ^*−*^flies. Ovaries from *sim-bam-yfp; bam* ^*−*^flies were stained with antibodies to Vasa (red) and Hsp-60 (green), which recognizes *Wolbachia*. *Wolbachia* preferentially accumulate at the somatic stem cell niche (arrow) of the germarium. Scale bar, 50μm.

Female *sim-bam-yfp; bam* ^*−*^flies have reduced fertility (Fig 3B). We therefore compared the fertility of *mel-bam-yfp; bam* ^*−*^and *sim-bam-yfp; bam* ^*−*^females with and without *Wolbachia* (*w*Mel) and found that *mel-bam-yfp; bam* ^*−*^fertility was neither enhanced nor diminished in the presence of *Wolbachia*. In contrast, we found a significant increase in the fertility of young *sim-bam-yfp; bam* ^*−*^females (days 1–5) infected with *Wolbachia*, a result which was consistent across multiple insertion sites (Fig 9A).

If *Wolbachia* has co-evolved with *bam*, one possibility is that *Wolbachia* levels will be influenced by the species-specific ortholog of *bam* that is present in females. To test this, we used qPCR to measure *w*Mel *Wolbachia* titer in ovaries and found that *Wolbachia* levels are reduced in *sim-bam-yfp; bam* ^*−*^compared to *mel-bam-yfp; bam* ^*−*^ovaries (Fig 9B).

One possible explanation for this reduced titer is that *Wolbachia* does not localize properly in *sim-bam-yfp; bam* ^*–*^. While *Wolbachia* is present in low levels throughout the germarium, it preferentially accumulates in the somatic stem cell niche (SSCN) in *D. melanogaster [46,48,49]*. As germline cysts pass the SSCN, high *Wolbachia* titer and prolonged exposure via somatic cells that encapsulate the cyst may allow it to efficiently infect the cyst and ensure vertical transmission [48]. We examined *Wolbachia* accumulation using an antibody to Hsp60 which cross-reacts with *Wolbachia* [44,59,60]. We found that as in *mel-bam-yfp; bam* ^*–*^, *Wolbachia* accumulates normally within the SSCN in *sim-bam-yfp; bam* ^*−*^flies (Fig 9C).

## Discussion

### Using transgenic rescue to identify divergent functions of adaptively evolving genes: Utility and caveats

A detailed comparison of *bam* function in *D*. *melanogaster* versus *D*. *simulans* is not possible due to the lack of available *bam* mutations in *D*. *simulans*. More importantly, such an approach might be insensitive to functionally important amino-acid changes if compensatory mutations have occurred in other genes in either lineage. We therefore designed a transgenic construct of *D*. *simulans bam* and transformed it into *D*. *melanogaster*, along with a parallel *D*. *melanogaster* control construct transformed into an identical place in the genome using the phiC31 transformation system [61].

We designed our constructs to have more non-coding DNA than a previously used *bam* transgene [21,23], yet found that both sets of *D*. *melanogaster bam* constructs have lower mRNA expression in females than a wildtype *bam* allele. Despite this expression difference, we found that our *mel-bam-yfp* construct fully rescues a *bam* null mutation in females. One possible explanation is that female flies are indifferent to large differences in *bam* levels. Alternatively, Bam protein levels may be controlled by a feedback loop that can compensate for differences in mRNA levels. This hypothesis is supported by the fact that differences in protein level between *mel-bam-yfp; bam* ^*−*^and *sim-bam-yfp; bam* ^*−*^genotypes are considerably smaller than the corresponding mRNA level differences (compare Fig 2A with 2D). We were not able to reliably quantify mRNA levels in males, but the inability of *mel-bam-yfp* to fully rescue male sterility suggests that it also under-expresses in males. If so, it would also suggest that males are more sensitive than females to lower levels of *bam* or that a feedback loop involving *bam* in females is not present in males.

Our goal in this study was to compare the effects of *bam* coding sequence divergence, and therefore we made the *sim-bam-yfp* construct using the untranslated regions (UTRs) and non-coding DNA from *D*. *melanogaster*, expecting that it would express similarly to *mel-bam-yfp*. Surprisingly, we found that *sim-bam-yfp* significantly overexpresses relative to *mel-bam-yfp*. One possible explanation is that *sim-bam* contains diverged regulatory sequences within its coding sequence or introns that affect transcription initiation. A second possibility is that these regions affect mRNA stability. Finally, it is possible that our *sim-bam-yfp* construct contains an intragenic incompatibility affecting mRNA stability between the *D*. *melanogaster* and *D*. *simulans* portions of its transcript. If true, then a *D*. *simulans bam* genomic transgene might have been more effective than a chimeric gene composed of sequences from both species. That alternative, however, is not a panacea because even genes that have similar expression levels between *D*. *melanogaster* and *D*. *simulans* can mis-express when placed in a foreign species due to "cis x trans" regulatory divergence [57,62,63].

We have performed several controls to minimize the complications arising from the differential mRNA expression levels of the *mel-bam-yfp* and *sim-bam-yfp* transgenes. First, we used the endogenous *D*. *melanogaster bam* locus as an additional control because its expression is not significantly different from *sim-bam-yfp* expression in ovaries (Fig 2A). Second, we have shown that the YFP protein localization patterns in both ovaries and testes are similar for both transgenes and resemble wildtype Bam (Figs 5A–5D, S2B and S2C).

We also note that female fertility levels do not appear to be highly sensitive to *bam* expression level. *mel-bam-yfp; bam* ^*−*^and the *bam* heterozygote are not significantly different in their levels of female fertility even though they express at different levels. Furthermore, *sim-bam-yfp* fertility rescue is significantly lower than both genotypes despite having a similar expression level to the *bam* heterozygote. These findings provide confidence in our conclusion that *sim-bam-yfp* has functionally diverged in its female germline function.

### *bam* divergence strongly affects female but not male functions

Reproductive genes are strongly affected by sexual selection, adaptive divergence, and intra- and inter-sexual conflict. Many lines of evidence suggest that these forces affect males more strongly than females. For example, hybrid male sterility evolves much more rapidly than hybrid female sterility, demonstrating that functionally relevant divergence between species is more likely to occur in males [64–66]. Gene expression of male-biased genes diverges more between species than does the expression of female-biased genes [67,68]. Finally, genes encoding male reproductive proteins are among the most rapidly evolving classes of genes [2,4,5,69,70].

GSC regulatory genes also are over-represented among adaptively evolving gene classes [7,14], which is surprising considering that there is no obvious role for sexual selection or sexual conflict to operate at such early stages of germline development. Selection to increase gamete production could occur in either sex, but would perhaps be stronger in males where energetic investment in gametes is less than for females. We were thus surprised to see how clearly *sim-bam-yfp* divergence affects female but not male fertility, even when males were assayed under stringent sperm exhaustion conditions.

Only in females does *bam* function in GSC differentiation [24,71]. Forced expression of a *bam* transgene in GSCs results in their differentiation only in females and not males [71]. Only after males are exposed to a longer duration and occurrence of heat shock are GSCs lost in males [72–74]. Instead, *bam*’s primary role in males is regulating cyst divisions and entry into meiosis [24,25,30]. Elegant studies have shown that increased or decreased levels of *bam* result in cysts with either less or more cells per cyst, respectively, which give rise to elongating spermatids and presumably mature sperm [25]. Therefore, males may be less affected by *sim-bam-yfp* divergence because either they are less sensitive to *bam* expression differences or females have additional sex-specific functions of *bam*.

In our fertility assays, we found that the *bam* trans-heterozygous mutants used in the female fertility assay resulted in reduced rescue in male fertility assays, presumably due to the accumulation of background mutations that affect male fertility (see Materials and Methods). Therefore, transgenic experiments in males were performed using a different combination of *bam* alleles. We consider it unlikely that the different allelic combinations underlie the sex-specific differences we see in the ability of *mel-bam-yfp* or *sim-bam-yfp* transgenes to rescue. *mel-bam-yfp* expression level in females is not significantly different in these two *bam* mutant combinations, arguing that the different genetic backgrounds do not cause a general difference in *bam* expression (Fig 2A).

### The molecular nature of *sim-bam-yfp; bam* ^*−*^defects

*sim-bam-yfp; bam* ^*−*^ovaries display a range of defects but never the "bag-of-marbles" phenotype seen in *D*. *melanogaster bam* loss-of-function mutations. The increased severity of phenotypes with increased *sim-bam-yfp* dosage also argues against a loss-of-function effect. Furthermore, the presence of *D*. *melanogaster bam* does not fully rescue *sim-bam-yfp; bam* ^*−*^defects, suggesting that it may have both loss and gain of function properties.

Since Bam and its interacting partner Bgcn are both adaptively evolving, we hypothesized that these ovarian defects might be due to an inability of sim-Bam to interact with mel-Bgcn. We provide three lines of evidence against this. First, *bgcn* is required for *bam*’s role in GSC differentiation. If this interaction were eliminated or reduced, we would expect to see tumorous ovaries but never do in *sim-bam-yfp; bam* ^*−*^flies. Second, sim-Bam::HA and mel-Bgcn::MYC reciprocally co-immunoprecipitate with one another in S2 cells. Third, removing one copy of *bgcn* does not exacerbate *sim-bam-yfp; bam* ^*−*^ovarian defects nor does it cause tumorous ovaries. This combination of biochemical and genetic data strongly suggests that *sim-bam-yfp; bam* ^*−*^defects are due to incompatibilities with *D*. *melanogaster* genes other than *bgcn*.

GSC loss is one of the most striking phenotypes we discovered in *sim-bam-yfp; bam* ^*−*^flies, a phenotype that was enhanced with additional copies of *sim-bam-yfp* transgenes (Fig 6). While *bam* is transcriptionally repressed in the GSC in wildtype *D*. *melanogaster*, there is a small amount of Bam protein present in the GSC which must be kept inactive (i.e. not cytoplasmic) [22,32,75]. One hypothesis to explain Bam silencing is that all Bam protein present in GSCs is localized at the spectrosome (i.e. round fusome), rendering it inactive in promoting differentiation [22,76]. This hypothesis is supported by data in which a subset of antibodies show Bam localized to the fusome. Bam itself is required for function of the fusome, and in *bam* mutants, the spectrosome shows a reduced amount of vesicular material [22]. A second hypothesis suggests that there is a small amount of cytoplasmic Bam present in the GSC, but that other proteins antagonize its activity [32,75]. Only after Bam accumulates to high levels can it titrate away antagonizing proteins and bind to other partners to promote differentiation.

Based on our data, we suggest that *sim-bam-yfp; bam* ^*−*^GSC loss results from sim-Bam-YFP either (1) failing to localize to the spectrosome, thus leaving it active in the GSC cytoplasm, and/or (2) preventing other proteins from localizing to the spectrosome. Fusome-protein components change during fusome growth and assemble in a hierarchical manner [27,76,77]. Based on our dominance study, we also hypothesize that the fusome cannot properly form in *sim-bam-yfp; bam* ^*−*^ovaries but can when *D*. *melanogaster bam* is added, thus allowing proper fusome localization of sim-Bam-YFP and/or other proteins. We favor this hypothesis since *sim-bam-yfp* flies also show mitotic synchrony defects, a hallmark of improper fusome function. Moreover, proper endocytic recycling of the fusome is required for GSC maintenance, as *rab11* mutants show GSC loss and have defects similar to *bam* mutants [77]. We have been unable to fully test this model though as Bam-F antibodies which show fusome localization [22] are no longer available and anti-Gfp antibodies used with our *bam-yfp* transgenes do not show fusome localization (see Fig 5), a result seen previously with different epitope-tagged transgenes [21].

### *Wolbachia* increases the fertility of *bam* mutant genotypes

Although *bam* is essential for fertility of both sexes, we only detected fertility defects in female *sim-bam-yfp; bam* ^*−*^flies. We cannot of course exclude the possibility that an unexamined aspect of male reproduction is impaired; nevertheless, it seems highly implausible that *bam* divergence is being driven by a selective force operating in males if the functional consequences of that divergence are so clearly deleterious in females. We therefore sought to identify selection pressures that could potentially drive female-specific functional divergence of *bam*. We examined the bacterial endosymbiont, *Wolbachia pipientis*, due to its maternal transmission and its ability to manipulate the reproduction of the hosts that it infects [39,40]. We found that *Wolbachia* infection increases the fertility of two different *bam* mutant genotypes: *D*. *melanogaster bam* hypomorphs, and *sim-bam-yfp; bam* ^*−*^females. It might be unexpected for a *D*. *melanogaster* strain of *Wolbachia* to partially rescue the female fertility defects of *sim-bam-yfp*. However, *sim-bam-yfp* at least partially maintains many of the same functions of wildtype *D*. *melanogaster bam*: promoting GSC differentiation, regulating cyst divisions, and interacting with *bgcn*. Therefore, an interaction with *Wolbachia* could potentially be maintained as well. We did though find that a *D*. *melanogaster*-specific strain of *Wolbachia* cannot accumulate to high levels when only *sim-bam-yfp* is present, suggesting an incompatibility between *D*. *melanogaster Wolbachia* and *D*. *simulans bam*. The lower *Wolbachia* titer might also explain why the level of rescue seen in *sim-bam-yfp; bam* ^*−*^(Fig 9A) was not to the level seen in the *D*. *melanogaster bam* hypomorph (Fig 8A).

### *Wolbachia*, *bam* and *Sex lethal*

The gene *Sex lethal* (*Sxl*) is required for *bam*’s function in GSC differentiation [35]. Intriguingly, *Wolbachia* partially rescues the female sterility of *Sxl* mutants in *D*. *melanogaster*. This interaction is allele-specific, suggesting that suppression is unlikely due to a general increase in germline *Sxl* expression [41]. Additionally, microarray studies showed no significant increase in *Sxl* expression when infected with *Wolbachia* [42]. *Sxl* is expressed in both GSCs and cystoblasts, while *bam* expression is repressed in GSCs and is active in cystoblasts and mitotically-active cysts, though each requires the other to promote differentiation [34,35]. Therefore, it has been proposed that *Sxl* partners with newly-expressed Bam in cystoblasts to promote differentiation by antagonizing *nanos* and likely other genes required to maintain GSCs [34–36]. Since *bam* itself provides cell-type specificity [78], we suggest that the increased fertility of *Wolbachia*-infected *Sxl* mutants is a result of increased *bam* activity driving the differentiation of GSCs, rather than a direct effect on *Sxl* activity.

### Is *Wolbachia* driving *bam* divergence?

*bam* has experienced recurrent, adaptive evolution in both *D*. *melanogaster* and *D*. *simulans* [12,13]. There is evidence that current *Wolbachia* infections in *D*. *simulans* have been present for at least 8.8x10^5^ generations [79] and possibly predating the speciation of *D*. *simulans* and *D*. *sechellia*, which would be > 2.4x10^6^ generations (assuming 10 generations/year) [80]. For *D*. *melanogaster*, however, the association appears more recent, 2.2x10^4^-8.0x10^4^ generations [81,82]. Therefore it is difficult based on current evidence to propose that *Wolbachia* has been the sole driver of *bam* divergence for *D*. *melanogaster*. It is possible, however, that the species has experienced recurrent infections resulting in the replacement of old infections not currently sampled today. *Wolbachia* can provide fitness advantages to its hosts; for example viral pathogen protection in *Drosophila* [83–86]. Therefore fitness benefits combined with cytoplasmic incompatibility can result in rapid displacement of less beneficial *Wolbachia* strains, an observation that has been reported for both *D*. *melanogaster* and *D*. *simulans* [81,87–89].

We therefore propose two models for how an interaction with *Wolbachia* may have driven the adaptive evolution of *bam*, while acknowledging that other factors may also have contributed. The first model assumes a mutualistic interaction between *bam* and *Wolbachia* and is inspired by research on the parasitic wasp, *Asobara tabida*, where *Wolbachia* is required for oogenesis to occur properly [90,91]. Pannebakker et al. [92] proposed that the initial introduction of *Wolbachia* infection suppressed normal host apoptosis that occurs during oocyte production, causing the wasp to adapt by upregulating apoptosis. This response, while beneficial in the presence of *Wolbachia*, results in hyperactive apoptosis and oogenesis inhibition in its absence [92]. Thus in this host, *Wolbachia* has transitioned from facultative parasite to obligate mutualist. While the precise mechanism underlying the *Wolbachia* effect is unknown, *Wolbachia* infection in insects alters the expression levels of numerous RNAs and proteins [93–96]. Thus in *D*. *melanogaster and D*. *simulans*, initial introduction of *Wolbachia* may have changed *bam* expression. Because these expression changes could affect fertility, strong directional selection would then act on *bam* to restore its proper expression in the presence of the bacteria. The result would be a mutualistic interaction between *Wolbachia* and *Drosophila* where *Wolbachia* provides a constant benefit to host GSC differentiation.

Our second model assumes an antagonistic interaction between *bam* and *Wolbachia*. In the ovary, GSCs continually divide, and a host must receive cues such as nutritional status and age to balance GSC division rates and GSC differentiation throughout its lifetime [97–101]. As a reproductive parasite, *Wolbachia* is reliant upon host oogenesis for transmission and wants to ensure that oogenesis is continually occurring. One way in which *Wolbachia* may increase oogenesis is to override host cues and cause GSCs to continually divide and differentiate by increasing *bam* activity. *Wolbachia* may act either directly on *bam*, or indirectly on antagonists of *bam* activity or on downstream differentiation factors. However, having too much *bam* activity would be deleterious to the host, as forced expression of *bam* in GSCs results in premature GSC loss [71]. Therefore, the host would respond by limiting overactive *bam* activity caused by *Wolbachia* infection. This conflict between host and endosymbiont over *bam* activity could lead to an evolutionary arms race.

The first model predicts that *bam* RNA and/or protein levels would be different in the presence of *Wolbachia*. The second model makes at least two predictions. The first is that both host and endosymbiont proteins involved in this interaction would adaptively evolve. A second prediction of the antagonistic interaction model is that each *Wolbachia* strain will have coevolved with its species-specific *bam* ortholog and that the transmission success of *Wolbachia* will be reduced in the presence of a heterospecific *bam* ortholog.

We examined the predictions of each model. For model 1, we found no evidence of altered *bam* expression at the RNA level, but we were unable to examine protein levels. In examining the predictions of model 2, it has already been shown that *bam* is adaptively evolving in both *D*. *melanogaster* and *D*. *simulans* [12,13]. While we do not know which *Wolbachia* genes are responsible for this interaction, the *Wolbachia* genomes of *D*. *melanogaster* strains (*w*Mel) and *D*. *simulans* strains (*w*Ri) differ dramatically. Ankyrin-repeat-domain-containing genes have extensively diversified between the two strains [102,103], which is intriguing because ankyrin repeats are known to mediate protein-protein interactions [104]. Thus this divergence may allow the different *Wolbachia* strains to target different host molecules [103]. In examining the second prediction of model 2, we found that the titer of *D*. *melanogaster*-specific *Wolbachia* is reduced in *sim-bam-yfp; bam* ^*−*^ovaries. It is important to note that *sim-bam-yfp; bam* ^*−*^ovaries show a range of defects, and thus could have an altered *Wolbachia* titer due to cellular differences from the control strain rather than a specific interaction with *Wolbachia*. We specifically used young flies to minimize such effects, but are unlikely to have fully eliminated them.

Further support of model 2 comes from our experiments testing *Wolbachia-bam* interactions. First, we find evidence of *Wolbachia* increasing *bam* activity (either directly or indirectly) in the *bam* hypomorph experiment where, when *bam* is not fully active, *Wolbachia* infection results in increased *bam* activity and thus increased fertility. We would expect the host to try to limit *Wolbachia* manipulation of *bam* and find evidence of this in our transgenic experiments where, in *mel-bam-yfp; bam* ^*−*^flies (with wildtype fertility), *Wolbachia* infection is incapable of further increasing *bam* activity (i.e. no increase in fertility). Our data suggest that *D*. *melanogaster* has responded to *Wolbachia* manipulation by utilizing or perhaps developing a feedback system to regulate *bam* activity. The feedback structure limits the ability of *Wolbachia* to overactivate *bam* activity, thus limiting deleterious effects on the host while still allowing increased *bam* activity when beneficial to the host. It should be noted in regard to GSC differentiation that this interaction does not suggest that mutualism has been established because the wildtype host shows no decrease in fitness without *Wolbachia*. It is only under specific *bam* mutant conditions that we see a fitness benefit to the host. Such conditions are unlikely to be common in nature, thus limiting any fertility benefit of *Wolbachia* infection.

Overall, our data are more consistent with the predictions of model 2. We note, however, that the predictions of each are not mutually exclusive. While altered *bam* RNA/protein levels are a prediction of model 1, this prediction is not incompatible with model 2. Similarly, the predictions of model 2, adaptive evolution of the genes involved and incompatibilities between *Wolbachia* and host proteins are also consistent with model 1.

Our discovery of interactions between *Wolbachia* and *bam* from *D*. *melanogaster* and *D*. *simulans* suggests that *bam* and *Wolbachia* have been interacting (either mutually or antagonistically) for an extensive period. We speculate that this history of association of *Wolbachia* with *D*. *melanogaster* and *D*. *simulans* has had major consequences on the evolution of *bam* in these species. Furthermore, infection with germline parasites may explain the more widespread pattern of adaptive evolution of early acting germline development genes [7,12,14,105].

## Materials and Methods

### Drosophila stocks and *Wolbachia* infection

All stocks were cultured at room temperature on standard yeast-glucose media. The *bam* ^*Δ86*^, *bam* ^*BW*^, *bam* ^*BG*^, and *bgcn* ^*1*^ stocks are described in FlyBase [106]. The *bam* ^*Δ59*^ allele was generated through a P-element excision of *bam* ^*1*^ (D. McKearin, pers. comm.). We sequenced this allele and discovered that the excision deletes all but the 31 amino acids from the C-terminal end of the protein. All five stocks were kindly provided by Dr. Dennis McKearin (HHMI). All stocks (including CS, *y w*, and transgenic stocks described below) were confirmed to be free of *Wolbachia* infection by PCR using primers wsp81F/wsp691R [107]. The *w*Mel-infected strain of *D*. *melanogaster*, *w*; *Sp*/*CyO; Sb*/*TM6B* +*w*Mel, was kindly provided by Dr. Bill Sullivan.

The *w*Mel strain of *Wolbachia* was introgressed by crossing *w*Mel-infected females into *bam* ^*Δ59*^ */TM3*, generating *bam* ^*Δ59*^ */TM3* +*w*Mel. The *bam* ^*Δ59*^ */TM3* +*w*Mel stock was then cured of *Wolbachia* by feeding the flies on media supplemented with 0.03% tetracycline for three generations, generating *bam* ^*Δ59*^ */TM3* Tet. Females of the *bam* ^*Δ59*^ */TM3* +*w*Mel stock were then backcrossed to males of the *bam* ^*Δ59*^ */TM3* Tet strain for at least six generations to generate genetically similar backgrounds including the mitochondria.

### DNA constructs

#### *mel-bam-yfp* transgene

We amplified a 4.1 kb fragment from genomic DNA of the sequenced *D*. *melanogaster* strain, *y*; *cn bw*; *sp*, using primers 904 and 905 (S4 Table). This fragment contains approximately 1.7 kb upstream of the *bam* start codon and approximately 1 kb downstream of the stop codon. The PCR product was cloned into the pCR-Blunt II-TOPO (Invitrogen) vector to generate the plasmid p{mel-bam} and verified by sequencing. A three-piece fusion PCR strategy was used to incorporate a Yellow fluorescent protein (YFP) tag into the *bam* coding region at the C-terminus. Two products were amplified using p{mel-bam} as the template with the primer pairs 906/907 and 908/909. These products correspond to parts of the *D*. *melanogaster bam* sequence directly upstream and downstream of the native stop codon. The third product containing the YFP tag was amplified using p{*w* ^*+mC*^ UAS-Lhr::Venus = UAS-Lhr::YFP} as the template [108] with primer pair 910/911. All three products were gel-purified and used as templates for fusion PCR for 6 cycles, and then primer pair 906/909 was added to amplify the final product. The final product was cloned into pCR-Blunt II-TOPO, verified by sequencing, and. the insert subcloned into p{mel-bam} using *Nde*I and *Stu*I restriction enzymes to generate p{mel-bam-yfp}. The full-length insert was then subcloned into the transformation vector pCasper4\attB [57] using *Not*I and *Kpn*I restriction enzymes and verified by sequencing.

#### *sim-bam-yfp* transgene

The *bam* genomic region was amplified from *D*. *simulans w* ^*501*^ genomic DNA using the primer pair 904/891, cloned into the pCR-Blunt II-TOPO vector and sequenced completely. A three-piece fusion PCR strategy was used to incorporate both the *D*. *melanogaster* regulatory region and YFP tag simultaneously. Two products for fusion were amplified using p{mel-bam-yfp} as template with primer pairs 926/927 and 930/931, corresponding to the *D*. *melanogaster* 5’ region and the 3’ regulatory region including the YFP tag, respectively. The third product for fusion was amplified from p{sim-bam} using primer pair 928/929. The gel-purified products were used as templates for fusion PCR as described above using primers 926 and 931, and the fusion product was cloned into pCR-Blunt II-TOPO and sequenced. The insert was subcloned into p{mel-bam-yfp} using *Mfe*I and *Stu*I, generating p{sim-bam-yfp}. The full-length insert was then cloned into the *Not*I and *Kpn*I sites of pCasper4\attB, and verified by sequencing.

### Transgenic fly lines

ΦC31-mediated transformation was used to generate transformants in *D*. *melanogaster* [61] and was performed by Genetic Services, Inc. Correct integration was assayed using a PCR-based assay developed by Venken et al. [109]. For the attP40 site at cytological position 25C6, the primer pair 949/1125 was used to check docking-site specificity. We discovered that the attP16 stock contains at least two attP docking sites at unknown locations. Southern blots using a probe designed to the *white* locus present on p{Casper4}\attB were used to determine that p{mel-bam-yfp} and p{sim-bam-yfp} both integrated into the same attP site (S6 Fig). We refer to this attP site in the attP16 stock as attP16a. All transformants were then outcrossed for at least six generations to a *y w* strain that had been inbred for 10 generations, to make the genetic backgrounds similar.

### Fertility assays

All crosses were performed at room temperature (22–23°C). Prior to crossing all flies were aged for 2–3 days post-eclosion on media supplemented with yeast. In female fertility experiments, single transgenic females were crossed to two wildtype Canton S (CS) males. The trio of flies were transferred to a new vial every five days for a total of 15 days and then discarded. Progeny from each vial were counted for 8 days after the first flies eclosed. In male fertility experiments, single males were mated to two wildtype CS females as described above. In sperm exhaustion assays, single males were mated to two wildtype CS females. The males were aspirated without anesthetizing into new vials containing two fresh CS females every day for 5 days. The females remaining in the vial were transferred to a new vial every five days for 10 days, and fertility was assessed by scoring the number of progeny that eclosed over 8 days.

For female fertility assays, the transgenes were crossed into the *bam* mutant background *bam* ^*Δ86*^ */bam* ^*Δ59*^. For male fertility we found that use of *bam* ^*Δ86*^ */bam* ^*Δ59*^ resulted in reduced fertility of *mel-bam-yfp* flies relative to the *D*. *melanogaster bam* heterozygous control, suggesting that background mutations in these mutants reduce male fertility. It is also likely that combinations of *bam* ^*Δ86*^ or *bam* ^*Δ59*^ with *bam* ^*1*^, the chromosome from which they were derived, will share these background effects. Therefore, all male fertility experiments were done with the transheterozygous combination *bam* ^*Δ86*^ */bam* ^*BG*^, which are independently-derived mutations of *bam*. In this background we found no reduction of fertility of *mel-bam-yfp; bam* ^*−*^relative to the *D*. *melanogaster bam* heterozygote under normal fertility assays.

### Gut-microbiome-controlled female fertility assay

To control for effects of tetracycline treatment on the gut microbiome in the *bam* ^*Δ59*^ */TM3* Tet line, axenic versions of the *bam* ^*Δ59*^ */TM3* +*w*Mel and the *bam* ^*Δ59*^ */TM3* Tet lines were generated and the gut microbiota from conventional (i.e., non-axenic) *bam* ^*Δ59*^ */TM3* +*w*Mel males were introduced to both lines. To generate axenic lines, embryos (less than 18 hour old) from the *bam* ^*Δ59*^ */TM3* +*w*Mel and the *bam* ^*Δ59*^ */TM3* Tet lines were collected and dechorionated with 0.6% sodium hypochlorite. Sterile embryos were then seeded onto standard sterile yeast-glucose media. Embryos were allowed to develop into adults, and to ensure the lines were microbe-free, 5 adults from each line were homogenized and all were plated onto MRS agar [110]. Axenic flies of each line were allowed to mate for one generation. To introduce a homogenous population of gut microbiota to the two lines and to control for genetic background, axenic virgin females were backcrossed for three generations to conventional males of the *bam* ^*Δ59*^ */TM3* +*w*Mel line collected from a single bottle. BC3 virgin females were then crossed to conventional *bam* ^*BW*^ males to generate the *bam* ^*BW*^ */bam* ^*Δ59*^ hypomorphic genotype.

Fecundity of these *bam* hypomorphs with and without *Wolbachia* was then assayed as follows. Prior to crossing all flies were aged for 3 days post-eclosion. Single *bam* ^*BW*^ */bam* ^*Δ59*^ +*w*Mel or *bam* ^*BW*^ */bam* ^*Δ59*^ Tet females were crossed with two wildtype Canton S males. The trio of flies was removed from the vial after 6 days and adult progeny were counted every other day for a total of 8 days. To ensure that *Wolbachia* infection status was accurately maintained, each mated female was homogenized at the end of the experiment and *Wolbachia* status was assayed by PCR with primers designed to *wsp* (wsp440F/wsp691R) and *dprA* genes (dprA483F/dprA663R). Female fertility was only analyzed for females whose *Wolbachia* status was consistent with the status of the original stock as determined by typing with PCR.

### Quantitative RT-PCR

Flies were aged 2 days on media supplemented with yeast. Ovaries were dissected in 1XPBS and total RNA was isolated from 10 ovaries using Trizol reagent (Invitrogen) following the manufacturer’s protocol. Samples were treated with 20 units DNaseI at 37°C for 2 hours (Roche) and purified using RNeasy columns (Qiagen) following the manufacture’s protocol. cDNA was generated from 2μg of total RNA using the Superscript III First Strand Synthesis kit (Invitrogen) and oligo-dT primers following the manufacturer’s protocol. Quantitative RT-PCR was performed on a Biorad MyiQ cycler using iQ SYBR Green Supermix (Biorad). For *bam*, primer pair 1160/1170 amplified *bam* from both species with high efficiencies. For *rpl32*, primer pair 844/845 from Maheshwari and Barbash [57] was used. The standard curve method was used to estimate *bam* and *rpl32* levels. Three technical replicates were performed from at least three biological replicates for each sample.

### Quantitative PCR

To assay levels of *Wolbachia*, qPCR was performed on genomic DNA as in [44,111]. Females who eclosed on days 1–2 were aged on media supplemented with yeast for 2 days post-eclosion. DNA was isolated from 10 ovaries using phenol-chloroform extraction followed by 2 rounds of ethanol precipitation and rehydration in water.

For *Wolbachia*, primer pair wsp440F/wsp691R was used [111]. For *rpl32*, primer pair 844/845 was used. The standard curve method was used to estimate levels of each product. Three technical replicates were performed from at least three biological replicates for each sample.

### Co-immunoprecipitation experiments

*D*. *simulans bam* was amplified from *w* ^*501*^ ovarian cDNA using primers 662/661, cloned into pENTR/D-TOPO vector (Invitrogen), verified by sequencing, and recombined into destination vectors using LR-Clonase II (Invitrogen) following manufacturer’s directions. *D*. *simulans bam* was recombined into pAFHW containing both Flag and HA epitope tags (http://emb.carnegiescience.edu/labs/murphy/Gateway%20vectors.html). *D*. *melanogaster bam* in pAFHW and *D*. *melanogaster bgcn* in pAFMW were kindly provided by D. McKearin [33].

Combinations of pAFMW-Bam and pAFHW-Bgcn or empty vectors were co-transfected into Drosophila S2 cells, cells incubated for 3 days, and then lysed in lysis buffer (50mM Tris-HCl pH7.8, 150mM NaCl, 0.1%NP-40). Anti-HA (Roche, 3F10) or anti-Myc (Roche, 9E10) antibodies were conjugated to 50 μl of Protein G Dynabeads (Invitrogen) in 200ul of PBST (0.01% Tween 20) at 4°C overnight with rotation. Antibody-conjugated beads were then added to cell lysate (80μg total protein) in 200μl in lysis buffer containing 1X protease inhibitor (Roche) and 1mM PMSF and incubated at 4°C overnight. Washes were performed following manufacturer’s directions and Dynabeads were boiled in 1X SDS sample buffer to elute protein.

### Western blotting

25–35 ovaries from females aged 2–3 days post-eclosion on media supplemented with yeast were homogenized in lysis buffer (50 mM Tris-HCl pH 7.5, 10 mM EDTA, 1.25% TritonX-100, 1X protease inhibitor, Roche) and centrifuged at 14000 rpm at 4°C for 5 minutes. Total protein in the supernatant was estimated using the Bradford assay (Biorad) and samples were boiled in an equal volume of 4X SDS sample buffer for 5 minutes. 10–20 μg were loaded on 10% SDS-PAGE gels. Primary antibodies were anti-GFP Jl-8 (Clontech, 1:2000) and mouse anti-tubulin T5168 (Sigma; 1:120,000). Secondary antibodies were HRP conjugated goat anti-mouse (Jackson; 1:1,000 for anti-GFP and 1:60,000 for anti-tubulin) and were detected with ECL Western blotting substrate (Pierce).

### Immunostaining

Immunostaining was performed as in Aruna et al. [112]. Primary antibodies were: anti-GFP (Invitrogen A6544, 1:200), anti-vasa (DSHB, 1:25), anti-1B1 (DSHB, 1:4), monoclonal anti-Bam (1:100). Anti-Bam antibody was provided by D. McKearin. Secondary antibodies including goat anti-rat, anti-rabbit, or anti-mouse were conjugated with Alexa fluor dyes (Molecular Probes, 1:200–1:500). Samples were mounted in Vectashield containing DAPI (Vector Laboratories) and analyzed using the Leica SP2 confocal microscope at the Cornell University Core Life Sciences Microscopy and Imaging Facility. Images were resized in Photoshop (Adobe, version 11.0).

## Supporting Information

S1 Fig Diagram of male and female transgenic rescue crosses. A) Female transgenic crosses. Heterozygous *bam* ^Δ86^ females were crossed to transgene-containing males. Male progeny from cross #1 containing *bam* (identified by the non-Stubble (Sb) phenotype of *TM3*) and the transgene (identified by expression of its *w* ^*+*^ marker) were then crossed with *bam* ^Δ59^ heterozygous females. *bam* mutant females were identified by their ebony (e) and non-Stubble phenotype. If *Wolbachia* was assayed in an experiment, it was introduced at cross #2 through the *bam* ^Δ59^ mother. B) Male transgenic crosses. Heterozygous *bam* ^Δ86^ females were crossed to transgene-containing males. Male progeny from cross #1 containing *bam* and the transgene (identified as above) were then crossed with *bam* ^BG^ heterozygous females. *bam* mutant males were identified by their heterozygous ebony (e), darker eye color (two copies of *w*+), and non-Stubble phenotypes.
(TIF)

S2 Fig *bam* rescue and localization. A) Both *mel-bam-yfp; bam* ^*−*^and *bam-α; bam* ^*−*^males have increased sterility compared to a heterozygous control. Experiments were performed under sperm exhaustion conditions as in Fig 4B, except that the number of sterile males that produce no offspring is shown. Transgenes (not including *bam*-α) are in site attP40. N = 24–30 males at day 1. B, C) Bam-YFP localization in B) *mel-bam-yfp; bam* ^*−*^and C) *sim-bam-yfp; bam* ^*−*^testes resembles wildtype patterns [25]. Testes are from flies aged 3–5 days post-eclosion and stained with antibodies to Vasa (green), Hts-1B1 (red), and YFP (blue). Scale bar, 50μm.
(TIF)

S3 Fig 2x *sim-bam-yfp; bam* ^*−*^expression and localization are similar to wildtype. (A) The expression of each transgene doubles with the addition of a second transgene copy. qRT-PCR of *bam* from ovarian mRNA from flies with 1 and 2 copies of *mel-bam-yfp* and *sim-bam-yfp*. *w* ^*1118*^ (brown) is shown as a wildtype reference. N = 3 biological replicates for each genotype. (*t*-test, *P<0.05). (B, C) sim-Bam-YFP localization resembles wildtype Bam localization even when multiple copies of *sim-bam-yfp* are present; two examples are shown. Ovaries are from flies aged 3–5 days post-eclosion and stained with antibodies to Vasa (green), Hts-1B1 (red), and YFP (blue). Scale bar, 50μm.
(TIF)

S4 Fig *sim-bam-yfp* rescues the tumorous phenotype caused by the *bam* hypomorph-*bgcn* interaction. (A) As described in Ohlstein et al. [31], removal of one copy of *bgcn* exacerbates a *bam* hypomorph resulting in completely tumorous ovaries. The egg chambers of these ovaries are filled with small nuclei. (B) The addition of one copy of *sim-bam-yfp* suppresses the tumorous ovary defects. Ovaries are stained with DAPI.
(TIF)

S5 Fig Fertility of gut-microbiota-controlled *bam* hypomorphs with and without *Wolbachia*. One gut-microbiota-controlled *bam* hypomorph female and two tester males from were allowed to mate and lay eggs for 6 days. The males were discarded and the females were assayed by PCR for final *Wolbachia* status. Fertility is reported as the average number of progeny per female +/- SEM (N = 7, *bam*-Tet, and N = 11, for *bam +w*Mel). *Wolbachia*-positive *bam* hypomorphs are significantly more fertile than the *Wolbachia*-negative *bam* hypomorphs (Exact Wilcoxon Mann-Whitney Rank-Sum Test, ***P = 6.285e-05). An Exact Wilcoxon Mann-Whitney Rank-Sum Test was used, as the data did not meet the standard assumptions for a *t*-test.
(TIF)

S6 Fig Southern blot identifies transgenes in the same insertion site. (A) Genomic DNA was digested with EcoRV. (B) Genomic DNA was digested with ClaI. Blots were incubated with a probe designed to *w*+ on pCasper4\attB. Below each blot is a schematic showing the location of the *w*+ probe (red bar), the restriction enzyme sites (orange), the location of the *attB* and *attP* sequences (boxes with B and P), and the approximate sizes of the digested fragments. The red box over the membrane highlights the diagnostic fragment used to determine shared integration sites. Lines *mel-bam-yfp* 29–1 and 20–2 as well as *sim-bam-yfp* lines 24–1 and 1–1 were all derived from integrations in the attP16 stock carrying multiple *attP* sites. Lines *mel-bam-yfp* 7–2 *and sim-bam-yfp* 21–1 were derived from integrations into attP40 in which only one *attP* site is present. Also run on the gels are the undocked attP16 line and *y w* and *w* ^*1118*^ into which the transgenic stocks had been crossed. These data show that *sim-bam-yfp* line 1–1 and *mel-bam-yfp* line 29–1 are integrated in the same *attP* site, termed attP16a, and that *sim-bam-yfp* 24–1 and *mel-bam-yfp* 20–2 are both in a distinct site termed attP16b. The attP16a integrants were used in this study. These data also confirm that *mel-bam-yfp* 7–2 and *sim-bam-yfp* 21–1 are in the same insertion site, attP40.
(TIF)

S1 Table *sim-bam-yfp;bam* ^-^ flies have multiple ovarian defects. Ovaries were dissected from flies aged for 3–5 days post-eclosion on yeast. The difference between *mel-bam-yfp;bam* ^-^ and *sim-bam-yfp;bam* ^-^ is significant (P = 2.02 x 10^−5^, F.E.T., calculated at http://vassarstats.net).
(DOCX)

S2 Table GSC number in transgenic lines. Ovaries were dissected from flies aged for 3–5 days post-eclosion on yeast. N > 47 ovarioles for each sample.
(DOCX)

S3 Table *Wolbachia* genetically interacts with *D*. *melanogaster bam*. Ovaries from *bam*-Tet or *bam* +*w*Mel females aged 3–5 days post-eclosion were stained with DAPI and the number of egg chambers with nurse-cell positive nuclei were scored. F.E.T. *P* = 9.5e-4.
(DOCX)

S4 Table Primers used in this study. (DOCX)
