# Supplementary figures and images for: The Drosophila bag of marbles Gene Interacts Genetically with Wolbachia and Shows Female-Specific Effects of Divergence
Source: PLoS Genet. 2015 Aug 20;11(8):e1005453. doi: 10.1371/journal.pgen.1005453 (PMC4546362; doi:10.1371/journal.pgen.1005453)

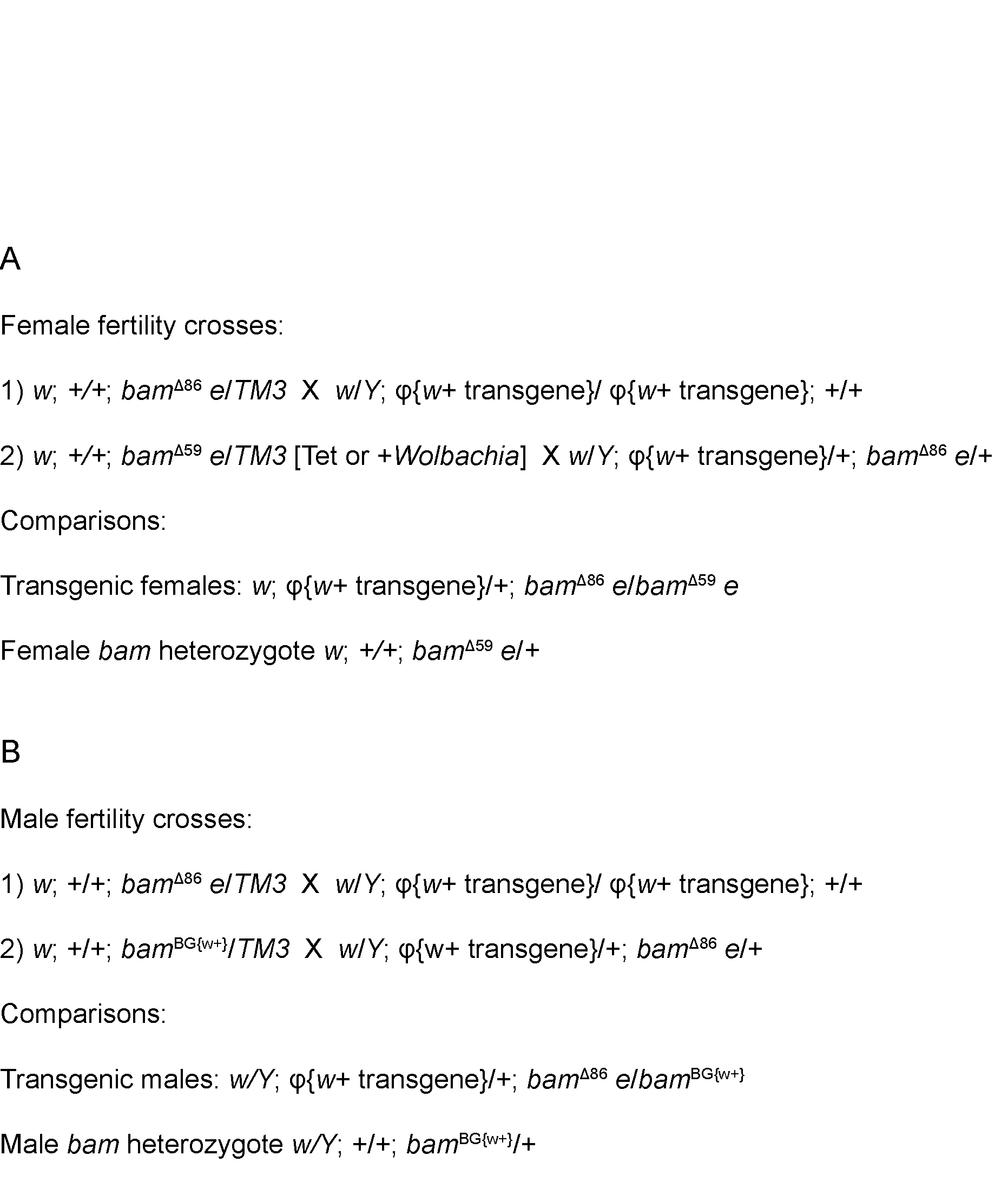

Supplement: S1 Fig — A) Female transgenic crosses. Heterozygous bam Δ86 females were crossed to transgene-containing males. Male progeny from cross #1 containing bam (identified by the non-Stubble (Sb) phenotype of TM3) and the transgene (identified by expression of its w + marker) were then crossed with bam Δ59 heterozygous females. bam mutant females were identified by their ebony (e) and non-Stubble phenotype. If Wolbachia was assayed in an experiment, it was introduced at cross #2 through the bam Δ59 mother. B) Male transgenic crosses. Heterozygous bam Δ86 females were crossed to transgene-containing males. Male progeny from cross #1 containing bam and the transgene (identified as above) were then crossed with bam BG heterozygous females. bam mutant males were identified by their heterozygous ebony (e), darker eye color (two copies of w+), and non-Stubble phenotypes. (TIF) [file pgen.1005453.s001.tif]

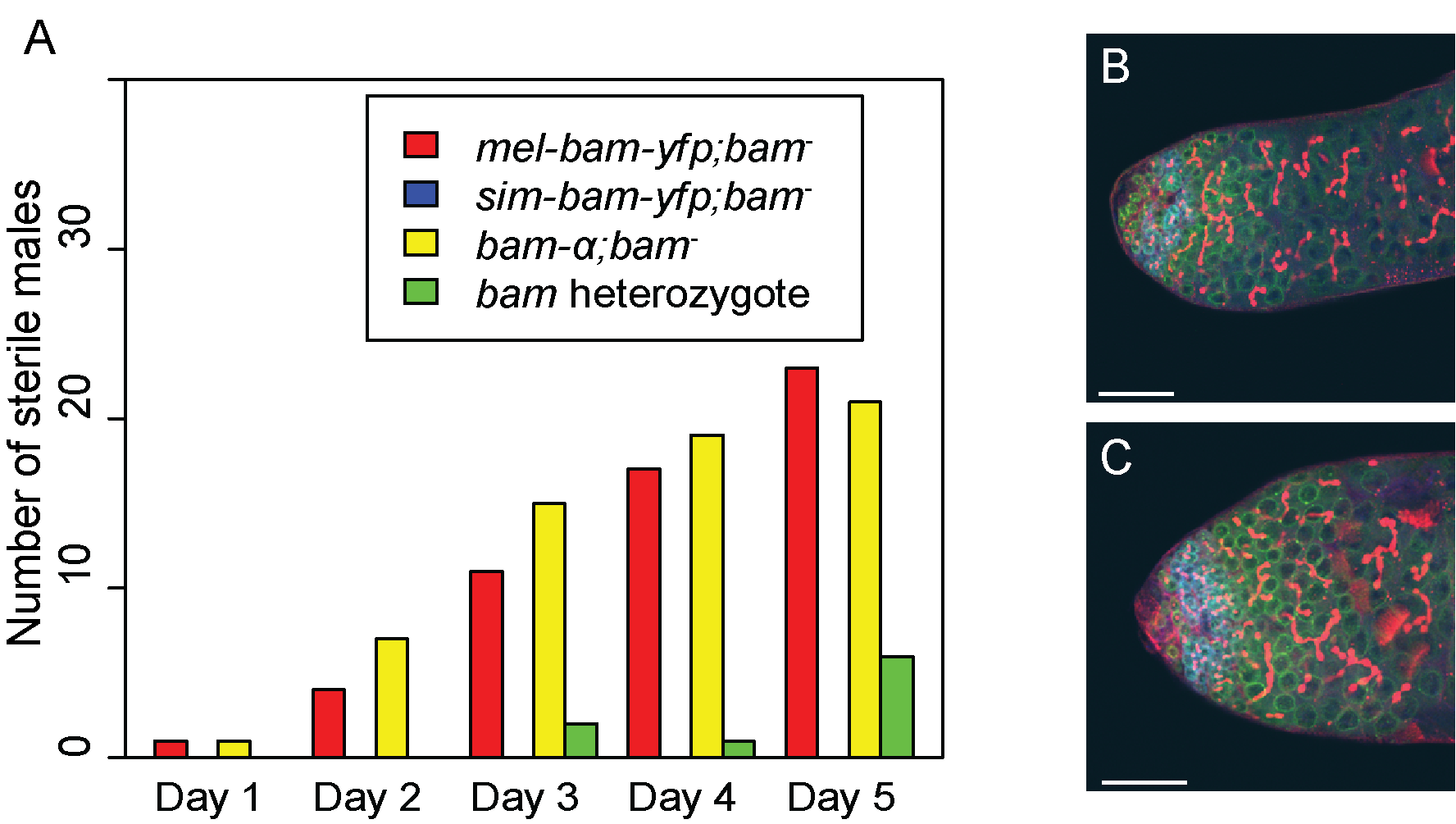

Supplement: S2 Fig — A) Both mel-bam-yfp; bam −and bam-α; bam −males have increased sterility compared to a heterozygous control. Experiments were performed under sperm exhaustion conditions as in Fig 4B, except that the number of sterile males that produce no offspring is shown. Transgenes (not including bam-α) are in site attP40. N = 24–30 males at day 1. B, C) Bam-YFP localization in B) mel-bam-yfp; bam −and C) sim-bam-yfp; bam −testes resembles wildtype patterns [25]. Testes are from flies aged 3–5 days post-eclosion and stained with antibodies to Vasa (green), Hts-1B1 (red), and YFP (blue). Scale bar, 50μm. (TIF) [file pgen.1005453.s002.tif]

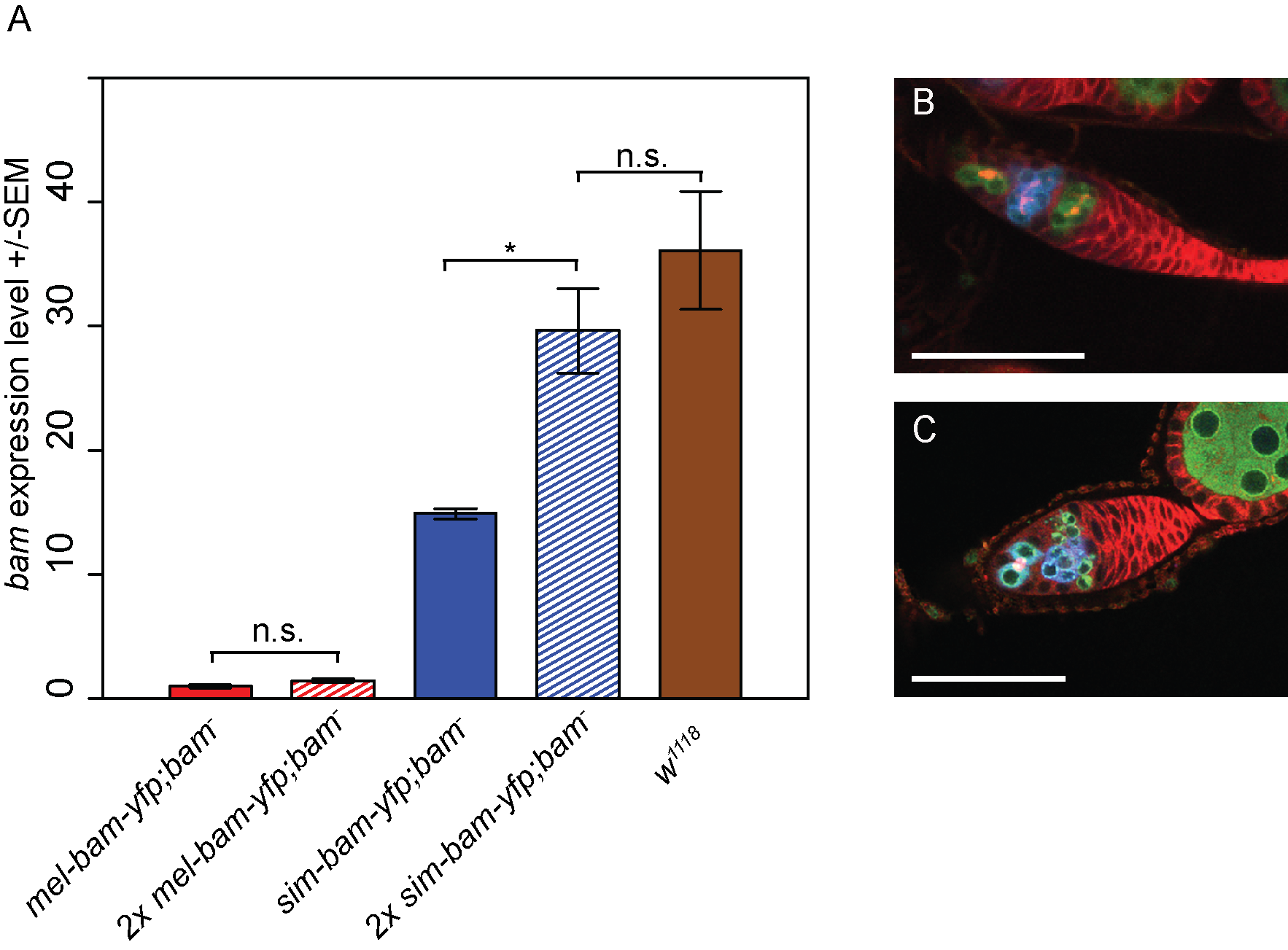

Supplement: S3 Fig — (A) The expression of each transgene doubles with the addition of a second transgene copy. qRT-PCR of bam from ovarian mRNA from flies with 1 and 2 copies of mel-bam-yfp and sim-bam-yfp. w 1118 (brown) is shown as a wildtype reference. N = 3 biological replicates for each genotype. (t-test, *P<0.05). (B, C) sim-Bam-YFP localization resembles wildtype Bam localization even when multiple copies of sim-bam-yfp are present; two examples are shown. Ovaries are from flies aged 3–5 days post-eclosion and stained with antibodies to Vasa (green), Hts-1B1 (red), and YFP (blue). Scale bar, 50μm. (TIF) [file pgen.1005453.s003.tif]

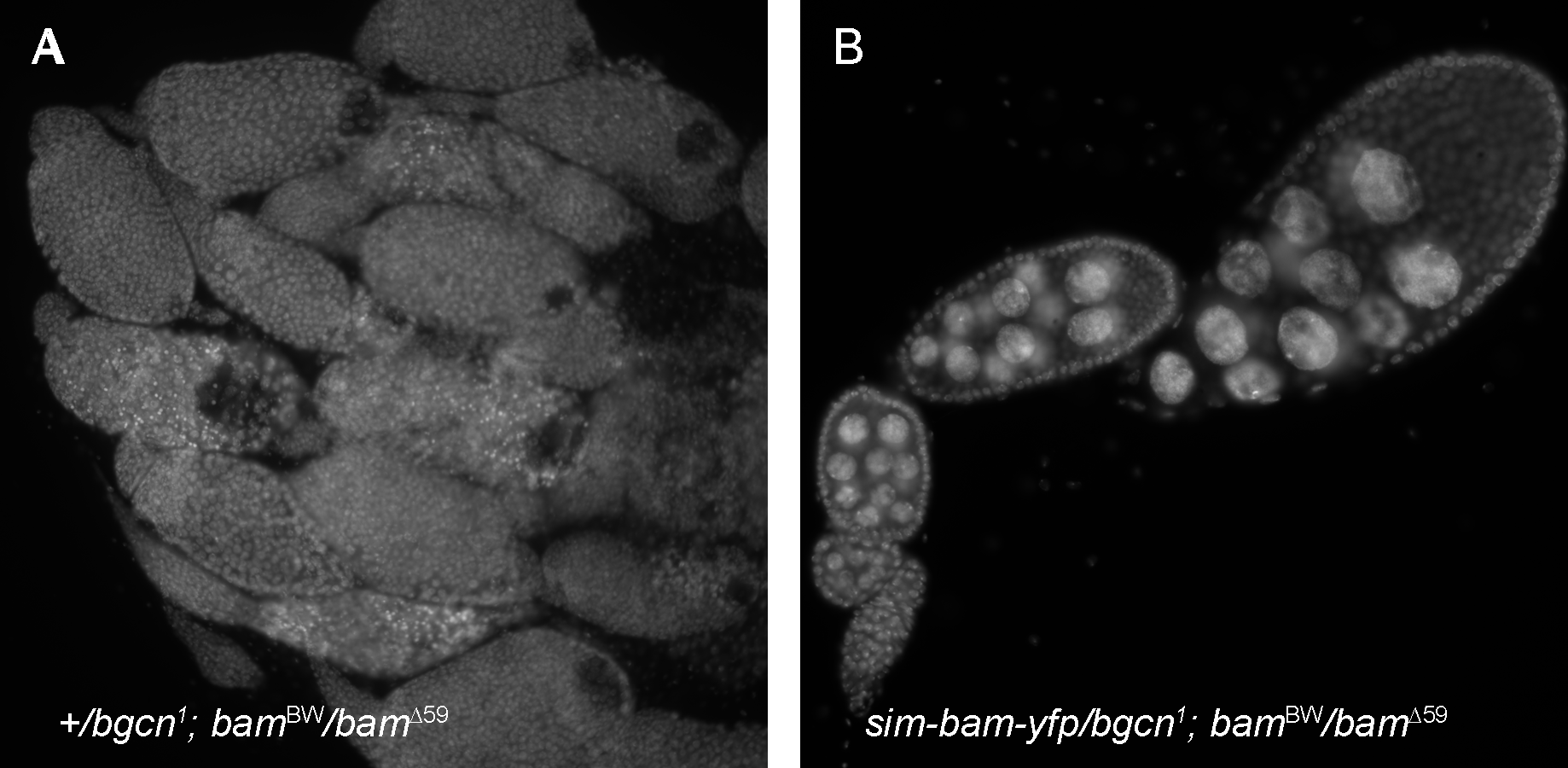

Supplement: S4 Fig — (A) As described in Ohlstein et al. [31], removal of one copy of bgcn exacerbates a bam hypomorph resulting in completely tumorous ovaries. The egg chambers of these ovaries are filled with small nuclei. (B) The addition of one copy of sim-bam-yfp suppresses the tumorous ovary defects. Ovaries are stained with DAPI. (TIF) [file pgen.1005453.s004.tif]

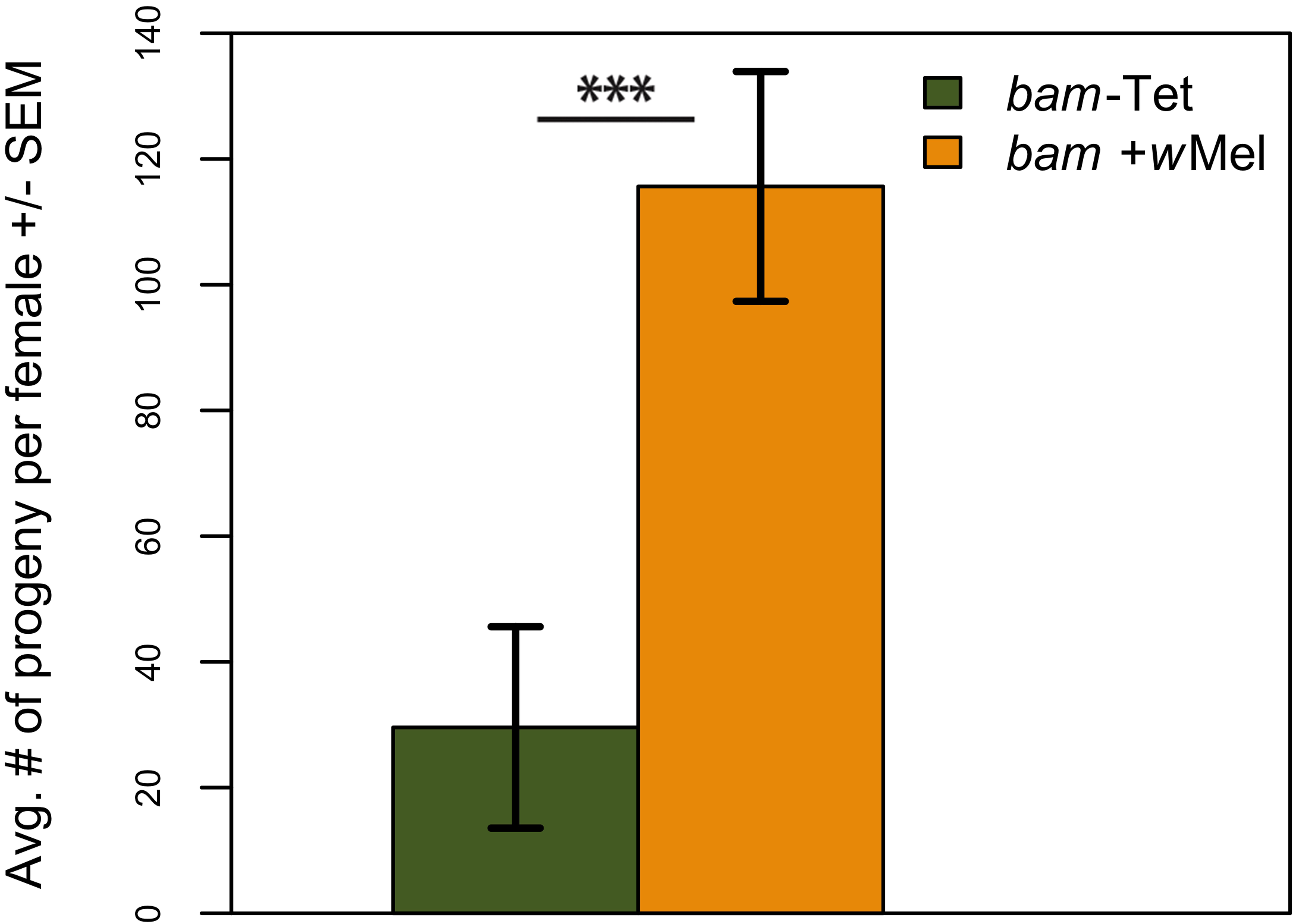

Supplement: S5 Fig — One gut-microbiota-controlled bam hypomorph female and two tester males from were allowed to mate and lay eggs for 6 days. The males were discarded and the females were assayed by PCR for final Wolbachia status. Fertility is reported as the average number of progeny per female +/- SEM (N = 7, bam-Tet, and N = 11, for bam +wMel). Wolbachia-positive bam hypomorphs are significantly more fertile than the Wolbachia-negative bam hypomorphs (Exact Wilcoxon Mann-Whitney Rank-Sum Test, ***P = 6.285e-05). An Exact Wilcoxon Mann-Whitney Rank-Sum Test was used, as the data did not meet the standard assumptions for a t-test. (TIF) [file pgen.1005453.s005.tif]

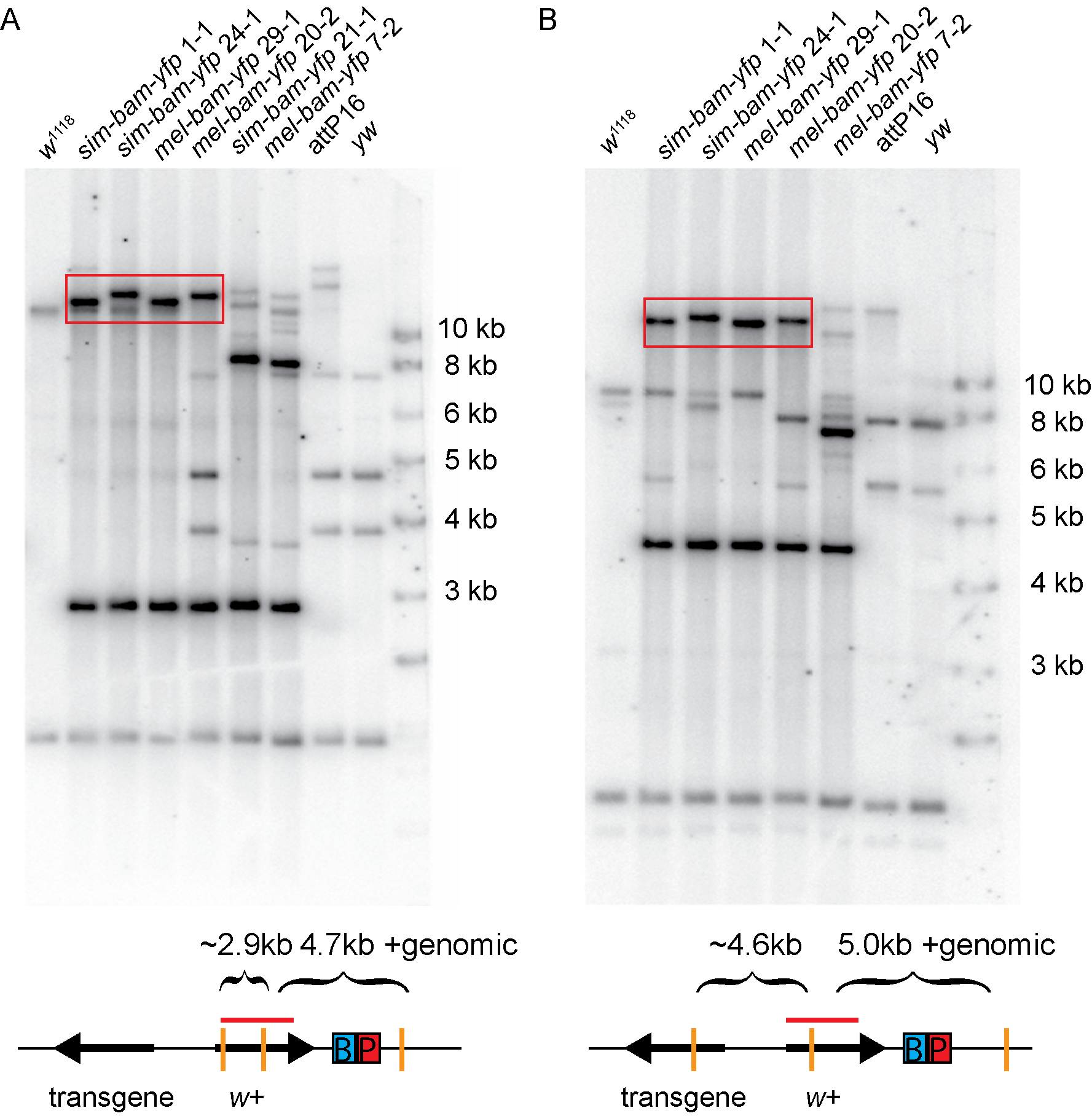

Supplement: S6 Fig — (A) Genomic DNA was digested with EcoRV. (B) Genomic DNA was digested with ClaI. Blots were incubated with a probe designed to w+ on pCasper4\attB. Below each blot is a schematic showing the location of the w+ probe (red bar), the restriction enzyme sites (orange), the location of the attB and attP sequences (boxes with B and P), and the approximate sizes of the digested fragments. The red box over the membrane highlights the diagnostic fragment used to determine shared integration sites. Lines mel-bam-yfp 29–1 and 20–2 as well as sim-bam-yfp lines 24–1 and 1–1 were all derived from integrations in the attP16 stock carrying multiple attP sites. Lines mel-bam-yfp 7–2 and sim-bam-yfp 21–1 were derived from integrations into attP40 in which only one attP site is present. Also run on the gels are the undocked attP16 line and y w and w 1118 into which the transgenic stocks had been crossed. These data show that sim-bam-yfp line 1–1 and mel-bam-yfp line 29–1 are integrated in the same attP site, termed attP16a, and that sim-bam-yfp 24–1 and mel-bam-yfp 20–2 are both in a distinct site termed attP16b. The attP16a integrants were used in this study. These data also confirm that mel-bam-yfp 7–2 and sim-bam-yfp 21–1 are in the same insertion site, attP40. (TIF) [file pgen.1005453.s006.tif]
