# Supplementary material for: The Drosophila bag of marbles Gene Interacts Genetically with Wolbachia and Shows Female-Specific Effects of Divergence
Source: PLoS Genet. 2015 Aug 20;11(8):e1005453. doi: 10.1371/journal.pgen.1005453 (PMC4546362; doi:10.1371/journal.pgen.1005453)
Supplement: S1 Table — Ovaries were dissected from flies aged for 3–5 days post-eclosion on yeast. The difference between mel-bam-yfp;bam - and sim-bam-yfp;bam - is significant (P = 2.02 x 10^−5^, F.E.T., calculated at http://vassarstats.net). (DOCX) [file pgen.1005453.s007.docx]

Table S1: *sim-bam-yfp;bam^-^* flies have multiple ovarian defects

|  |  | # of egg chambers with indicated # of cells/cyst | | |
| --- | --- | --- | --- | --- |
| Genotype | Transgene  Dose | <16 | 16 | >16 |
| *mel-bam-yfp;bam^-^* | 1 | 2 | 97 | 1 |
| *D. melanogaster bam* heterozygote | 0 | 1 | 96 | 3 |
| *sim-bam-yfp;bam^-^* | 1 | 10 | 76 | 14 |
