# Supplementary material for: The Drosophila bag of marbles Gene Interacts Genetically with Wolbachia and Shows Female-Specific Effects of Divergence
Source: PLoS Genet. 2015 Aug 20;11(8):e1005453. doi: 10.1371/journal.pgen.1005453 (PMC4546362; doi:10.1371/journal.pgen.1005453)
Supplement: S2 Table — Ovaries were dissected from flies aged for 3–5 days post-eclosion on yeast. N > 47 ovarioles for each sample. (DOCX) [file pgen.1005453.s008.docx]

Table S2: GSC number in transgenic lines

| Genotype | Mean # of GSCs per germarium |
| --- | --- |
| 2x *mel-bam-yfp;bam^-^* | 2.88 |
| 2x *sim-bam-yfp;bam^-^* | 0.5 |
| 2x *sim-bam-yfp;bam/+* | 2.33 |
