# Supplementary material for: The Drosophila bag of marbles Gene Interacts Genetically with Wolbachia and Shows Female-Specific Effects of Divergence
Source: PLoS Genet. 2015 Aug 20;11(8):e1005453. doi: 10.1371/journal.pgen.1005453 (PMC4546362; doi:10.1371/journal.pgen.1005453)
Supplement: S3 Table — Ovaries from bam-Tet or bam +wMel females aged 3–5 days post-eclosion were stained with DAPI and the number of egg chambers with nurse-cell positive nuclei were scored. F.E.T. P = 9.5e-4. (DOCX) [file pgen.1005453.s009.docx]

Table S3: *Wolbachia* genetically interacts with *D. melanogaster bam*

| Genotype | # of nurse cell-positive egg chambers | # of ovarioles examined |
| --- | --- | --- |
| *bam* Tet | 350 | 190 |
| *bam* +*w*Mel | 372 | 126 |
