# Supplementary material for: The Drosophila bag of marbles Gene Interacts Genetically with Wolbachia and Shows Female-Specific Effects of Divergence
Source: PLoS Genet. 2015 Aug 20;11(8):e1005453. doi: 10.1371/journal.pgen.1005453 (PMC4546362; doi:10.1371/journal.pgen.1005453)
Supplement: S4 Table — (DOCX) [file pgen.1005453.s010.docx]

Table S4. Primers used in this study

| No. | Sequence |
| --- | --- |
| 661 | TTAGCTTCTGAAGCGAGGTACAC |
| 662 | CACCATGCTTAATGCACGTGACATG |
| 844 | CCGCTTCAAGGGACAGTATC |
| 845 | GACAATCTCCTTGCGCTTCT |
| 904 | GCAAAAGATCTTCTGCACCCTCTG |
| 905 | TTTAGCGGATTCACAAGGGATCTC |
| 891 | TTTAGCGGATTCACAAGTGATCTC |
| 906 | CTGCATATGATTGGTCTGCACGG |
| 907 | AGCTCCTCGCCCTTGCTCACCATGCTTCTGAAGCGAGGTACACGTCC |
| 908 | TCTCGGCATGGACGAGCTGTACAAGTAAACTAATGCTGTGCACATCGATA |
| 909 | CCAGAAAGATCTCAGCGAGAACATG |
| 910 | GGACGTGTACCTCGCTTCAGAAGCATGGTGAGCAAGGGCGAGGAGCT |
| 911 | TATCGATGTGCACAGCATTAGTTTACTTGTACAGCTCGTCCATGCCGAGA |
| 926 | CTCACTGTCCAATGTTCCTTC |
| 927 | CCCATGTCACGTGCATTAAGCATTATTCTTAAGTTAAATCACACAAATC |
| 928 | GATTTGTGTGATTTAACTTAAGAATAATGCTTAATGCACGTGACATGGG |
| 929 | GCTCCTCGCCCTTGCTCACCATGCTTCTGAAGCGAGGTACACGTATGG |
| 930 | CCATACGTGTACCTCGCTTCAGAAGCATGGTGAGCAAGGGCGAGGAGC |
| 931 | GCTGGAAAATCTGTTCAACGG |
| 949 | GTCGACGATGTAGGTCACGGTC |
| 1125 | CGCCTTGTCCAGTCCAAAAAG |
| 1169 | GCCCATAACTATTGAGAAACTGC |
| 1170 | GATCATGCAGGGATCTGAACAG |
| 1480 | CTGCTCCATGCTCACTGCGCCAAGCTTCTGTGACCCGCAAATGGCGAC |
| 1481 | GAGGAAGTGCCATCATCGCCACCTCGTCACACCATACGTGTACCTCGC |
| 1479 | GTCGCCATTTGCGGGTCACAGAAGCTTGGCGCAGTGAGCATGGAGCAG |
| 1482 | GCGAGGTACACGTATGGTGTGACGAGGTGGCGATGATGGCACTTCCTC |
| wsp81F | TGGTCCAATAAGTGATGAAGAAAC |
| wsp440F | CTGGTGTTAGTTATGATGTAAC |
| wsp691R | AAAAATTAAACGCTACTCCA |
| dprA483F | cagaacgtttcgatgcttca |
| dprA663R | tgttacagcaagcggaattg |
